# What are the limits to biomedical research acceleration through general-purpose AI?

**DOI:** 10.1038/s41598-025-32583-w

**Published:** 2026-01-12

**Authors:** Konstantin Hebenstreit, Constantin Convalexius, Stephan Reichl, Stefan Huber, Christoph Bock, Matthias Samwald

**Affiliations:** 1 Institute of Artificial Intelligence, Center for Medical Data Science, Medical University of Vienna, Vienna, Austria; 2 CeMM Research Center for Molecular Medicine of the Austrian Academy of Sciences, Vienna, Austria; 3 Accelerate Europe Initiative, Vienna, Austria

**Keywords:** Business and industry, Engineering, Health care, Medical research, Scientific community

## Abstract

Although general-purpose artificial intelligence (GPAI) is widely expected to accelerate scientific discovery, its practical limits in biomedicine remain unclear. We assess this potential by developing a framework of GPAI capabilities across the biomedical research lifecycle. Our scoping literature review indicates that current GPAI could deliver a speed increase of around 2x, whereas future GPAI could facilitate strong acceleration of up to 25x for physical tasks and 100x for cognitive tasks. However, achieving these gains may be severely limited by factors such as irreducible biological constraints, research infrastructure, data access, and the need for human oversight. Our expert elicitation with eight senior biomedical researchers revealed skepticism regarding the strong acceleration of tasks such as experiment design and execution. In contrast, strong acceleration of manuscript preparation, review and publication processes was deemed plausible. Notably, all experts identified the assimilation of new tools by the scientific community as a critical bottleneck. Realising the potential of GPAI will therefore require more than technological progress; it demands targeted investment in shared automation infrastructure and systemic reforms to research and publication practices.

**Supplementary Information:**

The online version contains supplementary material available at 10.1038/s41598-025-32583-w.

## Introduction

Artificial intelligence affects large parts of human society, yet its most profound impact may lie in **accelerating scientific discovery**. The Organisation for Economic Co-operation and Development recently emphasized that enhancing research productivity through AI could be “the most economically and socially valuable” application of this technology^1^, echoing Nobel laureate Robert Solow’s foundational insight that technological advancement—not merely capital or labor—drives sustainable economic prosperity^2,3^. Influential voices such as Nobel laureate Demis Hassabis and the World Economic Forum similarly underscore that the primary value of AI lies in accelerating science^4^.

AI models trained on specific tasks (‘narrow AI’) have long been used as part of biomedical research, including breakthroughs such as AlphaFold’s^5^ protein structure prediction capabilities. The advent of **general-purpose AI (GPAI)**, such as large language models (LLMs), marked a significant progression from narrow AI toward more generalized capabilities. GPAI models have been defined as exhibiting “significant generality and are capable of competently performing a wide range of distinct tasks"^6^.

Researchers face overwhelming numbers of scientific publications, massive data sets, and the increasing demands of multidisciplinary collaboration^7–11^. GPAI models **integrate knowledge across disciplines**, such as biology, chemistry, engineering, and computing^12^. They demonstrate proficiency **across diverse tasks**, including coding, mathematics, and logical reasoning—achieving or surpassing human-level performance on rigorous scientific and coding benchmarks^13,14^. These capabilities are enhanced by rapid progress in mixture-of-expert architectures^15^ and reinforcement learning^16^ techniques, leading to sophisticated **reasoning models** such as OpenAI’s GPT-5.1^17^, Google’s Gemini 3^18^, and Anthropic’s Claude Opus 4.5^19^. Finally, GPAI models can utilize **external tools** such as search systems (e.g., web search for scientific literature^20^, or programmatic access to literature, genomic and other major biomedical databases^21^, specialized narrow AI systems (e.g., RDKit^22^ for chemoinformatics^23^, or lab automation frameworks (e.g., autonomous protein engineering in self-driving laboratories^24^.

GPAI models also drive emerging **autonomous AI agents**, which independently plan, reason, utilize tools, and iteratively explore scientific problems, potentially without significant human oversight. Empirical evidence supports the transformative potential of agent-driven research acceleration^20,25–27^. AI agents quickly distill literature, recognize contradictions and develop new hypotheses^20,28,29^. Combined with continuously operating **self-driving laboratories**, research timelines for projects may shrink from years to months or weeks^30,31^. One notable example is GPAI-driven drug discovery for pulmonary fibrosis, where one company reported reducing the time from discovery to preclinical candidate from at least 5–6 years^32^ to 18 months^33^. This > 3× acceleration could accelerate the breaking of “Eroom’s Law”^34^, the trend of exponentially slower and more expensive drug development. However, while the acceleration of specific tasks is well-documented, the practical limits of the end-to-end research lifecycle remain unclear. For example, it is not yet understood how extreme efficiency gains in AI-driven tasks interact with the irreducible ‘non-compressible’ steps of biomedical research to determine the ultimate speed limit of scientific discovery.

It is important to distinguish GPAI-driven automation from traditional laboratory automation. Conventional laboratory automation increases throughput via pre-scripted, rigid workflows (e.g., liquid handlers executing a fixed protocol), but requires constant human supervision to design the experiment, interpret the data, and reconfigure the machinery for the next step. In contrast, GPAI systems introduce autonomy to the research process. By combining the ability to process unstructured information with decision-making capabilities, GPAI agents can not only execute tasks but also adapt to unexpected results, troubleshoot errors, and autonomously navigate the loop between cognitive planning and physical execution.

Our study estimates the potential acceleration of established biomedical research processes using GPAI within the current research paradigm. Rather than speculating about systemic changes such as replacing biological experiments with in silico alternatives (which remains difficult to predict and quantify) we focus on how GPAI can automate and accelerate research methods currently employed. This allows us to provide evidence-based, practical estimates of realistic acceleration factors that are relevant for the immediate future, while accounting for systemic or fundamental limitations inherent to biomedical research.

Within this scope, we explore the boundaries of biomedical research acceleration through GPAI, addressing three central questions:

1. What **acceleration factors** may current and future GPAI plausibly achieve across different biomedical research tasks?
2. What challenges and constraints might **limit** this acceleration?
3. How might GPAI **transform research workflows** and what are **key policy considerations** to ensure responsible innovation?

## Results

### Frameworks to assess the acceleration of biomedical research using GPAI

#### GPAI capability framework

To analyze the potential for GPAI-driven acceleration of biomedical research, we first developed a framework of GPAI capability levels. Drawing on multiple existing frameworks^35–38^ and analyzing biomedical research requirements, we synthesized a **simple**,** unified framework of GPAI research capability** with two key dimensions:

- **Cognitive capability**, encompassing research activities primarily involving information processing, analysis, and decision-making, including literature review, hypothesis generation, experimental design, data analysis, result interpretation, and manuscript preparation.
- **Physical capability**, involving laboratory procedures, experimental setup, and material handling, which are made possible by robotics, lab automation, and automated experiment execution.

For both cognitive and physical capabilities, we define **three levels** that chart the progression of GPAI integration into the research process:

- At the **“No GPAI”** level, humans perform all work manually, possibly assisted by non-GPAI tools.
- **“Next-level” capabilities** represent GPAI systems that partially automate the research cycle but require significant human intervention, as demonstrated by several current systems.
- **“Maximum-level” capabilities** represent a radically transformed future scenario with advanced capabilities and high-level autonomy where GPAI conducts expert-level research with minimal to no human supervision.

**Fig. 1:**
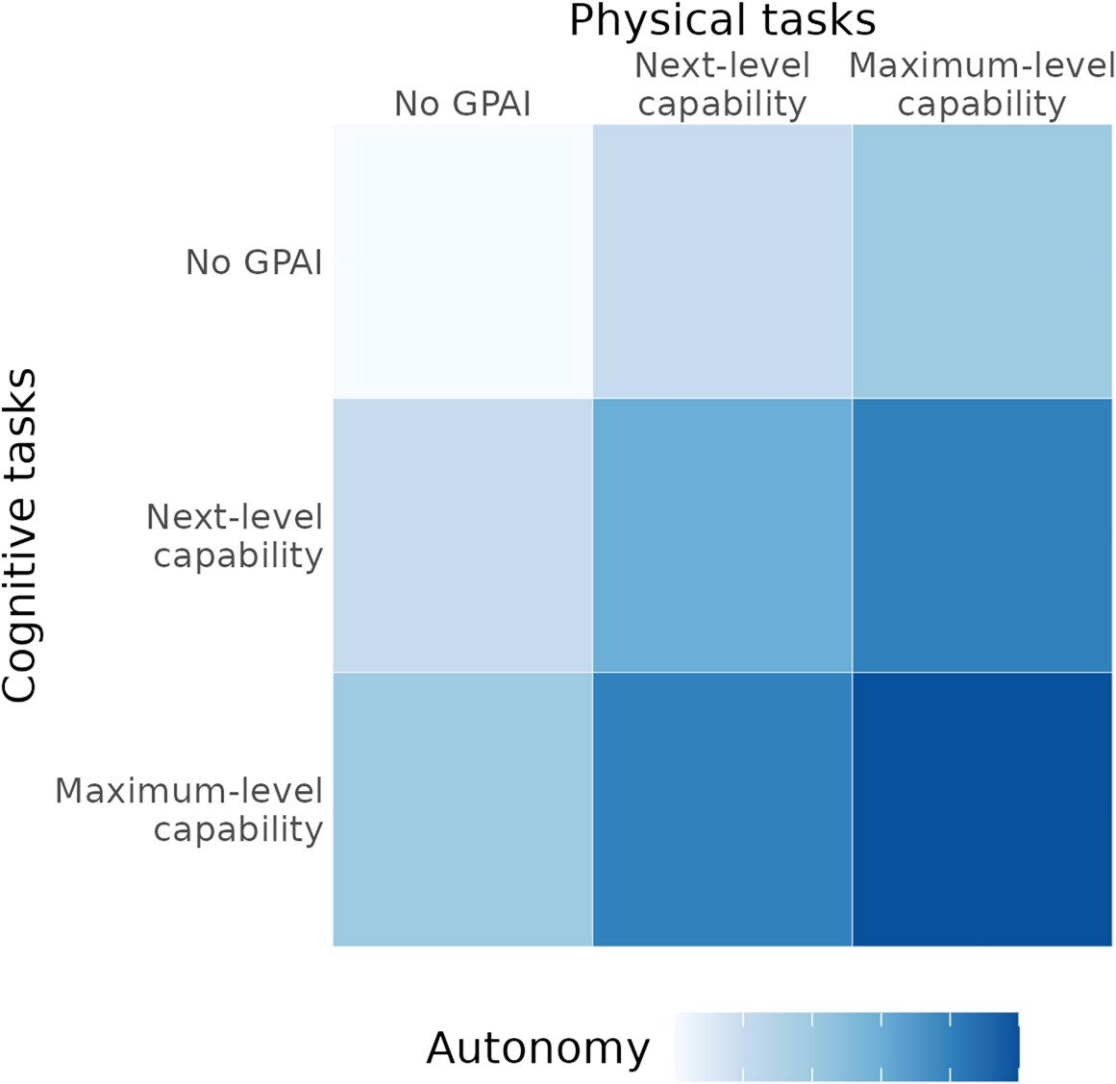
Capability framework illustrating how cognitive and physical capabilities of GPAI combine to yield autonomy. Higher autonomy levels enable increasingly significant acceleration of the biomedical research process. Adapted from Tom et al.^38^.

The joint development of cognitive and physical capabilities lead to increases in the emergent attribute of *autonomy*, i.e., the ability to operate independently across the complete research cycle. High levels of autonomy are key to the most radical acceleration of research (Fig. 1).

#### Research task framework

**Fig. 2:**
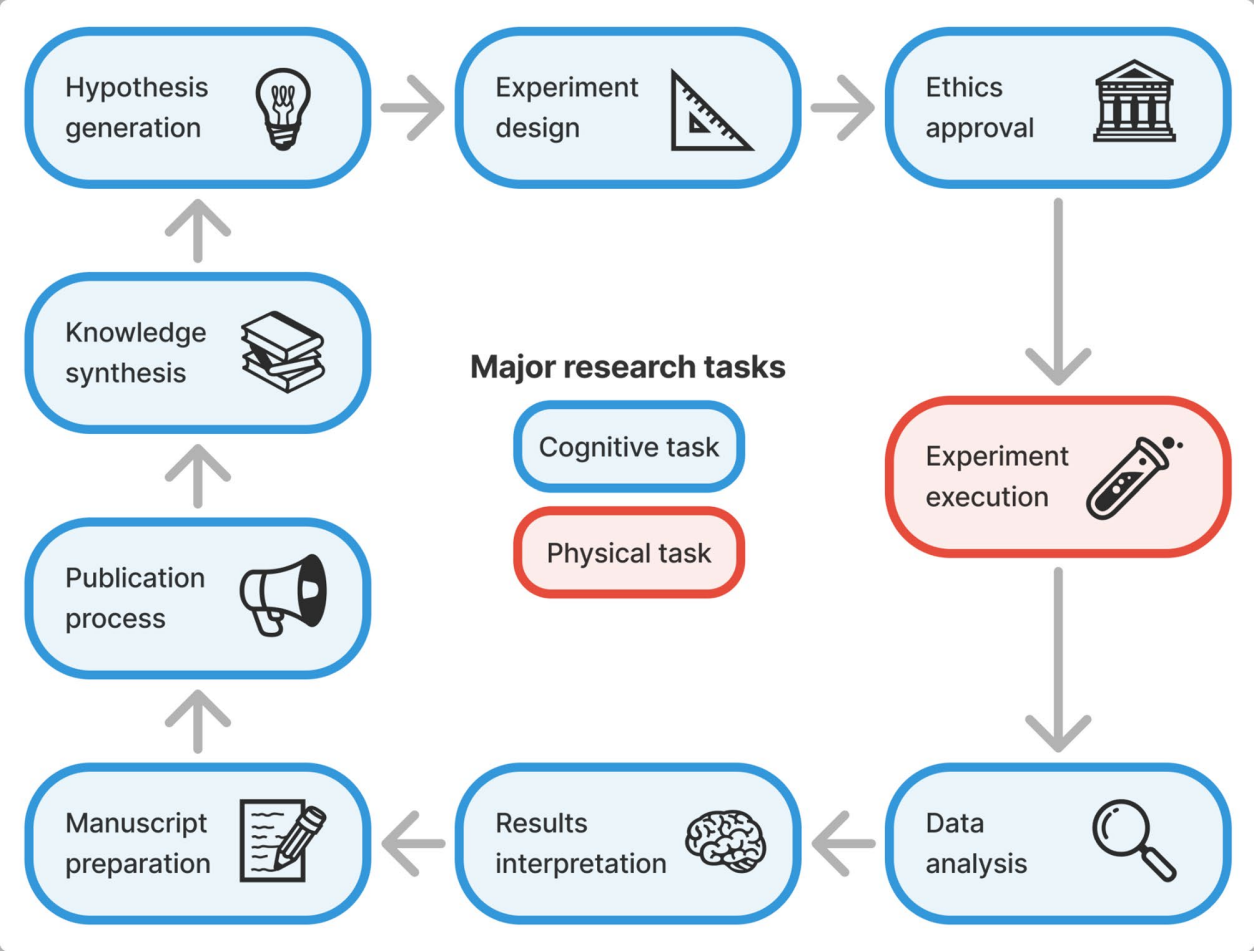
End-to-end biomedical research task framework covering nine major tasks. Blue boxes indicate cognitive tasks, the red box the sole physical task (experiment execution), and arrows depict the typical order and iterative nature of research projects.

Drawing on established concepts from the literature^39–43^, we developed a structured, end-to-end framework of the biomedical research process comprising nine major research tasks (Fig. 2), as well as constituent sub-tasks (Table S1). This allows us to map specific GPAI capabilities found in the literature to individual research tasks, track the varying levels of automation possible across different aspects of research, and better identify bottlenecks and opportunities for acceleration.

The major research tasks in our framework consist of the following:

1. **Knowledge synthesis** encompasses the collection, critical evaluation and integration of scientific information. Recent GPAI approaches achieve human-level or higher precision in literature-based tasks and operate at high throughput^29^. Systems automate literature search, evaluation, and summarization^21,44^ while demonstrating critical thinking and evaluation capabilities^27,45^.
2. **Idea & hypothesis generation** involves identifying promising research problems, formulating testable hypotheses, developing theoretical frameworks, and assessing feasibility. GPAI systems are increasingly able to develop new research ideas by analyzing patterns in existing literature and proposing candidate hypotheses and mechanisms^28,33,46^. Advanced systems can autonomously generate and, in some cases, rank candidate hypotheses by novelty and feasibility, then refine key research questions into actionable, experimentally testable goals^26,27,44,47^.
3. **Experiment design** includes method selection, protocol development, and planning data collection, including quality control. GPAI systems now demonstrate capabilities in suggesting experimental methods and designing wet-lab or computational protocols^27,28,48^, with advanced systems able to formulate experimental plans, predict and optimize parameters, and integrate quality control and reproducibility measures, including protocol standardization^21,24,44,49,50^.
4. **Ethical approval & permits** involve initial screening of research proposals, scientific review, ethics assessment, regulatory compliance, and administrative processing. While currently the most human-centered process, GPAI is beginning to assist in areas such as automated screening for ethical issues, documentation preparation, and compliance checks^51–53^. GPAI systems have potential to enhance informed consent processes^54,55^, improve scientific review efficiency, and support compliance checks against applicable regulations and guidelines^56^.
5. **Experiment execution** represents the physical dimension of research, including preparation, execution, documentation, troubleshooting, material management, and equipment maintenance. Self-driving laboratories can execute fully automated multi-step workflows with parallel samples, achieving significant acceleration through continuous operation, exception handling and adaptive optimization^24,30,57–59^. Advanced robotic systems execute protocols, monitor experimental conditions, and manage samples while improving throughput and reducing time requirements^24,31,60,61^.
6. **Data analysis** encompasses collection, cleaning, statistical analysis, visualization, and validation. GPAI systems increasingly automate much of the analysis pipeline, from preprocessing to statistical modeling and interactive exploratory analysis^49,62,63^. Modern systems collect and integrate diverse data types and automate data cleaning and preprocessing, with high agreement with expert analyses within end-to-end automated workflows^21,27,44,64^.
7. **Results interpretation** involves synthesizing experimental findings, evaluating hypotheses against results, and contextualizing findings within broader scientific knowledge. GPAI systems can now integrate computational and experimental data to connect predictions with outcomes and employ multi-agent approaches to critically evaluate results^21,27,28^. Advanced systems can compare experimental results to hypotheses and contextualize findings within existing knowledge^29,44^.
8. **Manuscript preparation** includes documenting methods, presenting results, managing references, creating figures and tables, facilitating data sharing, and handling writing and revision. GPAI systems can now generate complete manuscript drafts with methodological descriptions and iteratively improve them through automated feedback^45,65^. Modern systems document experimental methods, manage references, generate figures, and prepare shareable data and code^26,29,44,49,66^.
9. **Publication process** involves journal selection, submission, screening, peer review, and revision. GPAI assistance is emerging in areas such as journal and reviewer recommendation, formatting for submission, and generating peer reviews^67–70^. GPAI-generated feedback approximates human reviewers, and editorial experiments have found AI reviews sufficiently accurate to be helpful; GPAI can identify several (though not all) mathematical and conceptual errors^71–73^.

Each of these main tasks comprises several subtasks with varying potential for GPAI acceleration. While these main tasks usually proceed in the order presented, research projects often require iterative transitions between tasks, such as when feedback from reviewers requires a return to experimental design or when unexpected results necessitate revisiting hypothesis generation. Notably, experiment execution represents the physical dimension of research, while all other tasks are primarily cognitive. This framework forms the basis for our investigation of how different levels of GPAI capabilities can accelerate the biomedical research process.

#### Integration of GPAI capability and research task frameworks

Combining the frameworks introduced above yields a matrix of research acceleration scenarios across GPAI capabilities and major research tasks (Table S2).

This integrated framework allows us to systematically analyze how different levels of GPAI capability transform the research process, identify where the greatest potential for acceleration exists, and what bottlenecks might remain even with advanced GPAI systems.

### Evidence for acceleration potential

To obtain concrete GPAI acceleration factors, we conducted a scoping review of literature on GPAI accelerating research. It reveals significant variation in potential speedups between cognitive and physical tasks, with cognitive tasks generally showing higher acceleration factors^21,24,29,60^. The evidence ranges from modest improvements to dramatic transformations in research timelines.

#### Acceleration of cognitive tasks

The strongest empirical evidence for cognitive task acceleration comes from recent applications of GPAI in research environments, though the measurement approaches and reported metrics vary across studies. For instance, recent industry reports estimate GPAI-driven research and development in drug discovery from initial research to the preclinical stage, yielding a **1.3-2x efficiency increase**, corresponding to a 25–50% reduction in cost and time^74^. Analysis of flow cytometry data in clinical immunology has been accelerated **2-4x** by GPAI, reducing the required time from 10 to 20 min to 5 min, while maintaining expert-level accuracy^62^.

Looking at related tasks outside the core of science, studies have reported a **1.3x speedup** in consulting tasks^75^, a **1.7x speedup** (40% time decrease) in professional writing tasks^76^, and a **2.2x speedup** (55% time decrease) for coding tasks, according to GitHub’s internal report on Copilot^77^.

The reported acceleration potential increases significantly with more advanced GPAI systems and setups optimized for automation. For example, in scientific knowledge synthesis, PaperQA2 demonstrated an **~ 75-300x speedup**, writing high-quality Wikipedia-style articles in 8 minutes^29^ (a task that human editors report taking 10–40 hours^78^. In bioinformatics, a GPAI agent capable of fully automated multi-omic analyses reportedly just required 5 min for the exemplary task of identifying differentially expressed genes between bulk RNA-seq samples^49^. Compared to the 10–12 hours^79,80^ reported by two bioinformatics facilities for the same type of task, this represents an approximate speedup of **~ 120-140x**.

Extending to complete research cycles, a GPAI agent team claimed to have developed SARS-CoV-2 nanobodies in a fraction of the time human researchers would have needed^27,81^. Another automated biomedical GPAI system called BioResearcher reported achieving a **~ 150-300x speedup** by completing full dry lab research cycles, from literature searches to the execution of computational experiments, in approximately 8 h versus the traditional 7–14 weeks^21^. Similarly, Sakana AI’s ‘AI Scientist’ demonstrated high efficiency through full automation in computer science research, capable of exploring research ideas in ~ 15 min (a rate of ~ 50 ideas in 12 h)^26^.

#### Acceleration of physical tasks

In laboratory settings, physical task acceleration also shows promising results, though generally with somewhat lower acceleration factors than purely cognitive tasks. An integrated robotic chemistry system achieved a **1.7x speedup** in synthesizing nerve-targeting agents compared to manual methods, completing the entire 20-compound library in 72 h instead of 120 h, reportedly with comparable quality^82^.

Protein engineering with integrated GPAI testing and feedback demonstrated a reduction in project duration from 6 to 12 to six months **(1-2x speedup)** in real-life testing (including shipping delays). The authors suggest that with better planning it could be reduced to two months **(3-6x speedup)** and in the best-case scenario of continuous operation to just 1–2 weeks **(~ 15-50x speedup)**^24^. A GPAI-driven microbial culturomics platform overcomes the variability of manual methods by using imaging to autonomously inform colony selection, yielding a more than **20x speedup** by achieving an isolation throughput of 2,000 colonies per hour in an integrated pipeline^60^. An automated materials discovery platform integrated ML screening, robotic synthesis, and characterization, reportedly reduced material sintering times from 2 to 6 h to 10 min **(12-36x speedup)** and was noted to reduce entire processes from hours or days to minutes^83^.

An automated chemical workflow handling 16 parallel samples conducted 688 experiments in 8 days. Compared to manual methods, which were estimated to take half a day per experiment, this represents a **~ 40x speedup**. In addition to these results, the study reported estimated acceleration factors of **~ 10x**-**100x** compared to conventional workflows, where the lower range corresponds to semi-automated methods and the higher end to manual approaches^30^. Self-driving laboratories that integrate robotics, additive manufacturing, and GPAI were projected to accelerate materials and molecular discovery by **10–100x** through combining gains from robotics (2x), active learning (5-20x), process intensification (up to 100x) and continuous operation (2–3 ×)^84^.

Based on internal industry data, one prominent cloud lab suggests its GPAI implementation enables a **2x speedup** in time-to-publication (from an average of 1.96 years to one year) and claims to generate publication-quality data **90x faster** by reducing traditional 3-month timelines to 24 hours^85^. In a notable anecdote, a PhD student reported replicating years of their previous project’s work in just one week using an automated robotics platform with 24/7 operation **(~ 100x speedup)**^86^.

#### Challenges and constraints of biomedical research acceleration

The empirical evidence reviewed above highlights the potential for GPAI to accelerate both cognitive and physical research tasks, with some studies demonstrating order-of-magnitude improvements. These impressive figures often reflect optimized scenarios or specific sub-tasks. Realizing such acceleration consistently across the entire research lifecycle is subject to various **practical**,** biological**,** infrastructural**,** and institutional constraints.**

One major category of reported constraints relates to the **implementation and operation of automated systems and self-driving laboratories**. Studies note that creating robust self-driving laboratories requires significant investment and complex integration of automated experiments with GPAI decision-making^87,88^. Even once operational, researchers report challenges in automated or cloud lab environments including remote troubleshooting difficulties, reduced experimental flexibility for exploratory research, and limitations in applicability for academic settings characterized by frequent directional pivots^89^. Comparing the effectiveness of different self-driving laboratories is also reportedly difficult due to challenges in defining standardized performance metrics that capture the nuances of diverse lab setups^88^. Finally, translating discoveries made in controlled self-driving laboratory environments to real-world applications faces hurdles related to storage stability, limited resources, and self-sufficiency without expert supervision^90^.

Another set of limitations highlighted in the literature concerns data and the GPAI models themselves. The performance of models central to GPAI and self-driving laboratories can be hampered by shortcomings in available data, such as the common lack of negative results or detailed metadata in published literature^91^. Building robust GPAI decision-making models often requires large, high-quality, information-rich datasets, the generation of which can be a bottleneck^92^.

Perhaps the most fundamental constraints reported are inherent biological and physical limits. While automation can speed up workflows like liquid handling or data acquisition, the underlying biological processes often have irreducible timescales^89^. Cell-based experiments, for instance, remain resource-intensive and subject to variability, with limits imposed by factors like maximum cell growth rates under specific conditions^92^. Self-driving laboratories optimize experiments around these biological components rather than altering their intrinsic limits^87,91^. Even the speeds of biochemical processes, like enzyme kinetics or protein folding, impose natural limits that automation cannot bypass^90^. Finally the complexity of biology also presents a challenge, as fully understanding and predicting cellular behavior remains difficult without comprehensive perturbation data, even when advanced models are utilized^89,92^.

Aside from the technical and biological hurdles, significant limitations arise from established social and institutional structures and processes. Although GPAI can accelerate certain tasks, the overall pace can be slowed by procedural delays inherent in the current academic system, as they may not scale readily with technological advancements. Ethics approval processes average between 50 and 138 days^93^. Similarly, publication faces substantial delays: preprint-to-publication averages 199 days^94^, with submission-to-publication times within journals ranging from 91 to 639 days^95^. The peer review process itself was reported to take 17 weeks in one study^96^, despite reviewers spending only about six hours per review in each round^97^.

These documented limitations, which have been identified in the literature alongside the potential for acceleration, suggest that achieving maximum theoretical acceleration across entire research workflows poses significant practical, technological, and ethical challenges. In fact, even beyond these operational and inherent limitations, rigorously assessing the extent of GPAI-driven acceleration itself presents methodological complexities. The interpretation of reported accelerations requires clear baseline values and system boundaries. Highly task-specific accelerations, such as GPAI agents that rapidly design nanobodies^27^ or automate the planning and coding of bioinformatics analyses^21,49^, are valuable but must be distinguished from reductions in overall project duration.

Assessing the acceleration of research driven by GPAI requires methodological rigor, as the perceived benefits depend on the chosen benchmark (e.g., humans, optimized laboratories, or state-of-the-art automation). For example, robotic systems accelerate workflows such as chemical synthesis^30^ or high-throughput screening^86^ primarily through parallelism and continuous operation, rather than pure task speed. Such throughput gains are significant, but must be compared with appropriate advanced baselines—not just sequential execution by humans—and must take into account fixed setup costs that can reduce the benefit of switching to automated workflows, especially in isolated or small-scale projects.

Similarly, the acceleration offered by GPAI in the computer-aided discovery of therapeutics^33^ or proteins^24^ can only be judged when the validation effort for GPAI-generated hypotheses is taken into account. Therefore, precise reporting standards are crucial, requiring transparency regarding benchmarks, system boundaries, the distinction between actual speed and throughput, and full consideration of all operating and validation costs in order to accurately assess the benefit of GPAI.

### Acceleration factors with GPAI levels

**Fig. 3:**
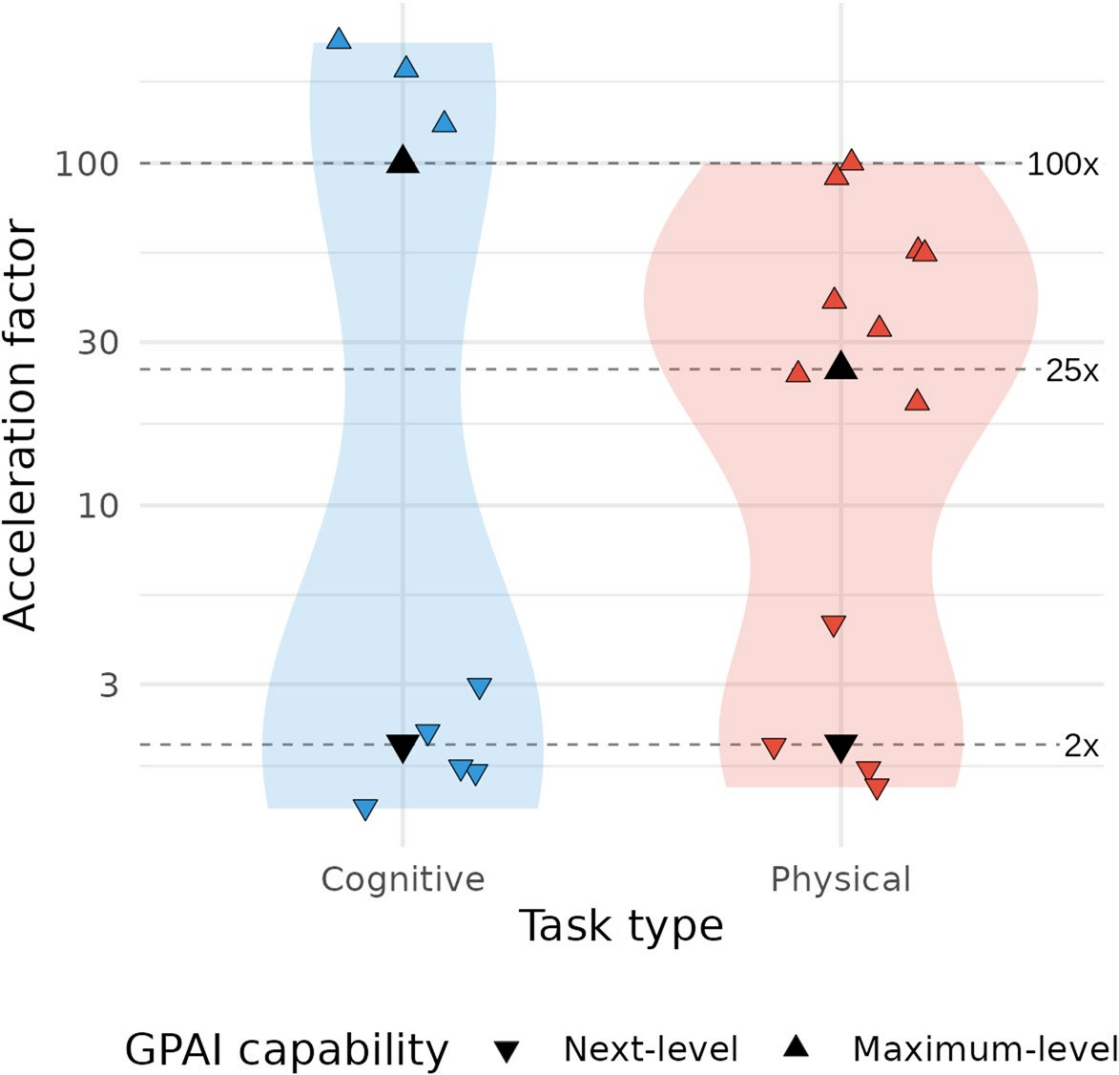
Reported acceleration factors for cognitive and physical research tasks. Violin plots depict the distribution of 20 acceleration factors on a log_10_-scale extracted from 16 publications for cognitive (blue) and physical (red) tasks. Individual studies are shown as jittered triangles; downward symbols (▼) indicate next-level capability, upward symbols (▲) indicate maximum-level capability. Four black triangles and three dashed lines (at 2×, 25×, 100×) denote the factors chosen for our modeling scenarios.

Our literature review reveals a bimodal distribution of acceleration factors across research tasks, with most observed values clustering either at lower levels (below 3x) or higher levels (above 10x for physical tasks and above 50x for cognitive tasks) (Fig. 3, Table S3). This pattern suggests two distinct regimes of reported GPAI-driven acceleration: incremental acceleration attainable today across the entire research process (Next-level GPAI), contrasted with transformative acceleration, achievable only through advanced systems and configurations (Maximum-level GPAI), and currently restricted to specific research tasks—thereby directing attention toward the theoretical maximum of research acceleration.

To translate these empirical findings into practical modeling scenarios, we assigned two distinct acceleration profiles to the capability levels defined in our framework:

1. **Next-level GPAI**: This profile models the **current acceleration potential** of current GPAI systems as they diffuse through the research ecosystem. Based on the lower cluster of our empirical findings, we estimate acceleration factors of **2× for both cognitive and physical tasks**—a mid-range value from observed current improvements (Fig. 3) We assume that these represent realistic, immediately achievable improvements that organizations can expect when implementing current GPAI and lab automation technologies, as already demonstrated in preclinical drug discovery for pulmonary fibrosis^33^.
2. **Maximum-level GPAI**: This profile explores the **transformative acceleration potential** of research with future, highly advanced GPAI systems. Drawing from the upper cluster of documented capabilities, we estimate acceleration factors of **100× for cognitive tasks** and **25× for physical tasks** (Fig. 3). We deliberately selected to err on the conservative side by picking acceleration factors from the lower end of reported values for physical tasks (where more empirical evidence exists) and below even the minimum observed value for cognitive tasks (where evidence is more limited). While these factors may seem extraordinary, they represent acceleration potentials that have been demonstrated in specific contexts and thus provide evidence for possible future scenarios.

#### Biological time constants in acceleration modeling

It is important to note that the acceleration factors cited above derive primarily from high-throughput in-vitro experiments and computational tasks. However, biomedical research, particularly involving whole organisms, contains certain irreducible processes that cannot be accelerated beyond natural biological limits (such as time for cell growth, animal model development, or tumor progression). We therefore include a “non-compressible” time constant in our model representing irreducible intervals dictated by biological processes that remain fixed regardless of technological advancement.

### Acceleration scenario modeling

Taken together, we propose the following simple formula to estimate research time:

**Total research time = (Compressible time ÷ Acceleration factor) + Non-compressible time**.

To demonstrate the effect of different acceleration scenarios on research time, we model a hypothetical 3-year biomedical research project representative of a typical PhD project duration. In this example we assume 24 months of cognitive work and 12 months of physical experimental work, of which 3 months represent biological time constants that cannot be compressed (Table 1).

**Table 1:** Worked example of GPAI-driven reduction in project duration for a hypothetical 36-month research project with a 3-month biological non-compressible time constant.

Project duration (in months)	Physical: No GPAI (9 + 3 = 12 months)	Physical: Next-level GPAI 2x-acceleration (9/2 + 3 = 7.5 months)	Physical: Maximum-level GPAI 25x-acceleration (9/25 + 3 = ~ 3.4 months)
Cognitive: No GPAI (24 months)	36.0 (= 1x)	31.5 (~ 1.1x)	27.4 (~ 1.3x)
Cognitive: Next-level GPAI 2x-acceleration (24/2 = 12 months)	24.0 (= 1.5x)	19.5 (~ 1.8x)	15.4 (~ 2.3x)
Cognitive: Maximum-level GPAI 100x-acceleration (24/100 = ~ 0.2 months)	12.2 (~ 3x)	7.7 (~ 4.7x)	3.6 (= 10x)

Cognitive tasks offer greater potential for GPAI-driven acceleration due to their longer initial durations and higher acceleration factors, lacking the inherent limitations of biological processes and requiring less infrastructural investment. Still, achieving the most significant acceleration across biomedical research depends on maximizing GPAI capabilities in both cognitive and physical domains.

Applying maximum-level GPAI to both cognitive and physical tasks in our example reduces a 3-year (36-month) biomedical research project to 3.6 months, a 10x acceleration despite the biological time constant. While this constant constitutes a small fraction of the total timeline without GPAI (3 of 36 months ~ 8.3%), it becomes the dominant factor (3 of 3.6 months ~ 83%) under maximum-level acceleration.

Fields heavily dependent on in-vitro or computational approaches may realize acceleration factors approaching our maximum estimates, while those requiring extensive in-vivo work will experience more modest overall timeline reductions due to the presence of larger biological time constants.

### Exploratory expert elicitation

In order to assess both the plausibility of our acceleration factors and limiting conditions, as well as the prevailing attitude and expectations of biomedical researchers toward GPAI for accelerating research, we conducted an exploratory elicitation with eight biomedical expert researchers. They (1) estimated time allocation across nine research tasks, (2) evaluated the plausibility of maximum-level acceleration factors (∼100× cognitive, ∼25× physical) for each task on five-point scales, (3) rated potential limiting factors on research acceleration, and (4) provided open-ended considerations.

**Fig. 4:**
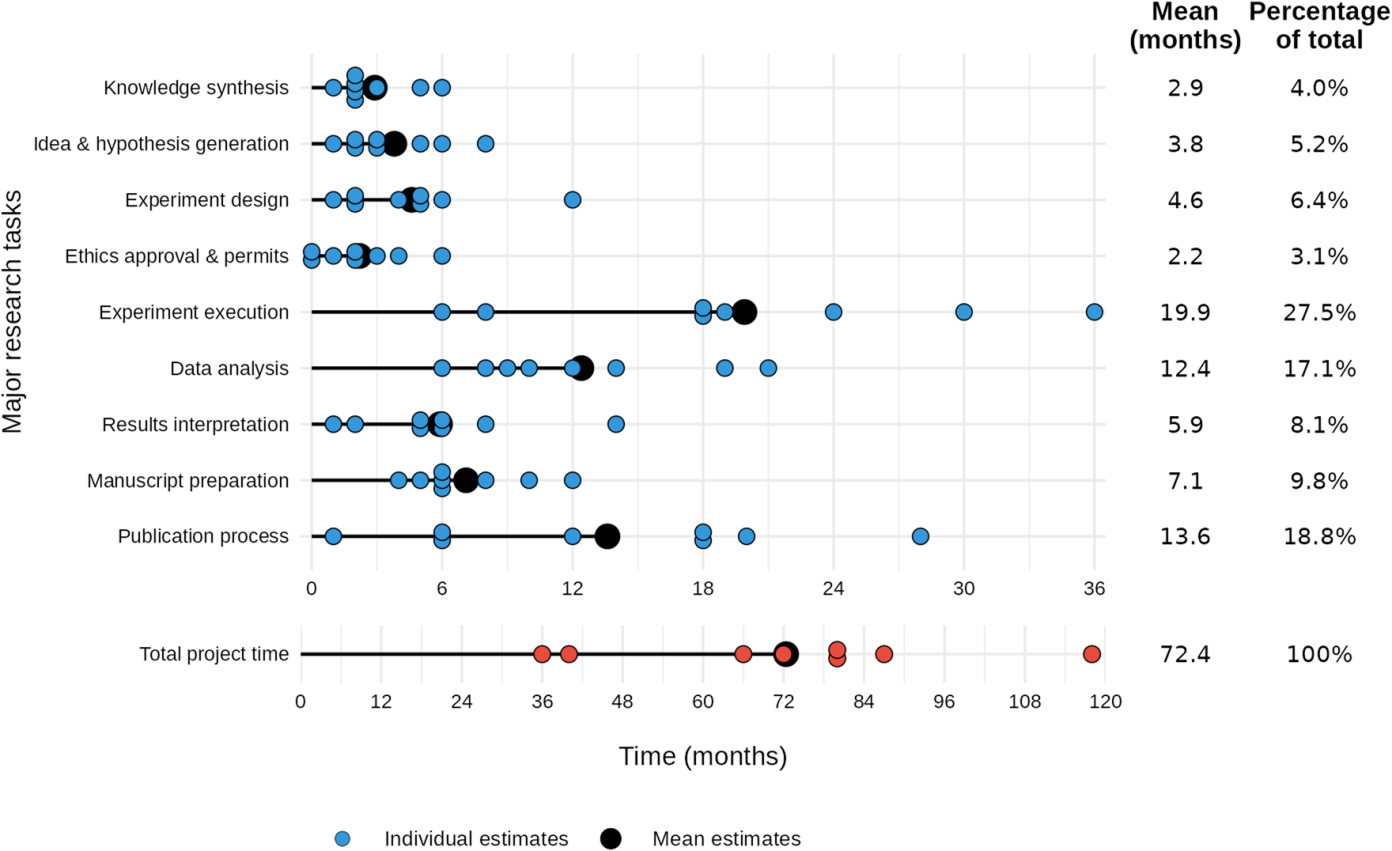
Timeline of major research task durations across studies. Horizontal dots show individual estimates (one dot per study) of time spent (in months, on a 6-month–interval axis) on each major research task, with lollipop-plots indicating the task’s mean. On the right, for each task, the rounded mean duration (months) and percentage of the total project time is shown. Below, a separate row presents individual and mean estimates for overall project duration (in months, on a 12‐month–interval axis).

The experts reflected on how our findings would apply to projects they had led from conception to publication in high-impact journals. They reported an average project duration of 72 months (Fig. 4), which is twice as long as our hypothetical example, but is consistent in the proportional distribution between cognitive and physical tasks: cognitive tasks took up 73% of project time (52 months), which is similar to the 67% in our hypothetical project. Experts identified “experiment execution,” “publication process,” and “data analysis” as the most time-consuming research tasks, while “ethics approvals and permits” and “knowledge synthesis” were rated as the least time-consuming.

**Fig. 5:**
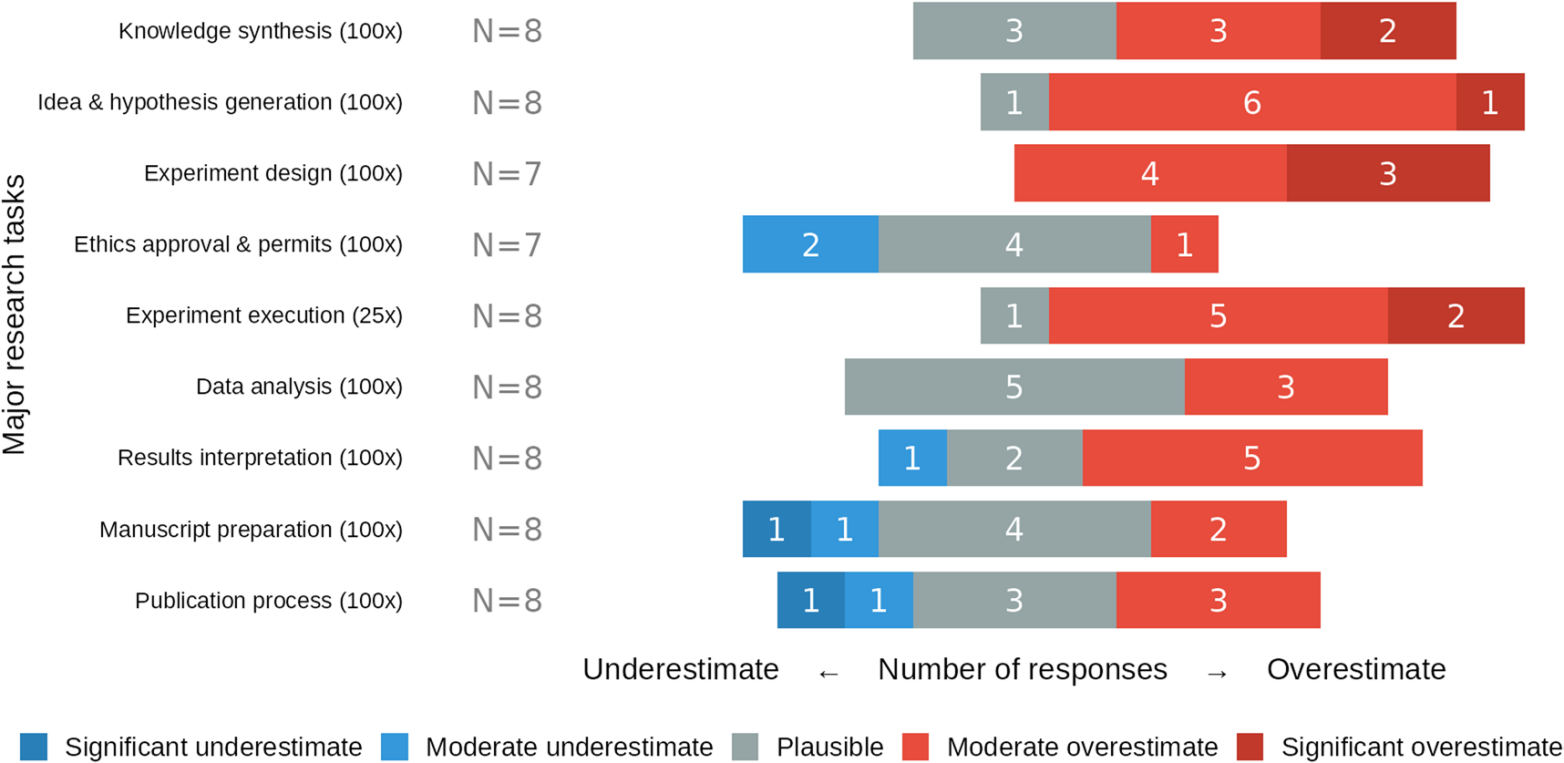
Perceived plausibility of maximum-level GPAI-acceleration factors across the nine major research tasks. Colors denote responses: significant underestimate (dark blue), moderate underestimate (light blue), plausible (grey), moderate overestimate (light red), and significant overestimate (dark red), with number of responses in white.

When **evaluating our estimates for maximum-level acceleration** (~ 100-fold cognitive, ~ 25-fold physical), biomedical experts judged experiment design and execution, and hypothesis generation to be strongly overestimated, while greater acceleration potential was deemed plausible for administrative tasks. Respondents consistently considered our acceleration estimates overestimated for experimental design (7/7 responses), experimental execution (7/8) and hypothesis generation (7/8). In contrast, experts considered high acceleration factors plausible for structured administrative processes: ethics approval (4/7), manuscript preparation (4/8), and publication processes (3/8), with the rest of the responses mixed between over- and underestimation (Fig. 5).

**Fig. 6:**
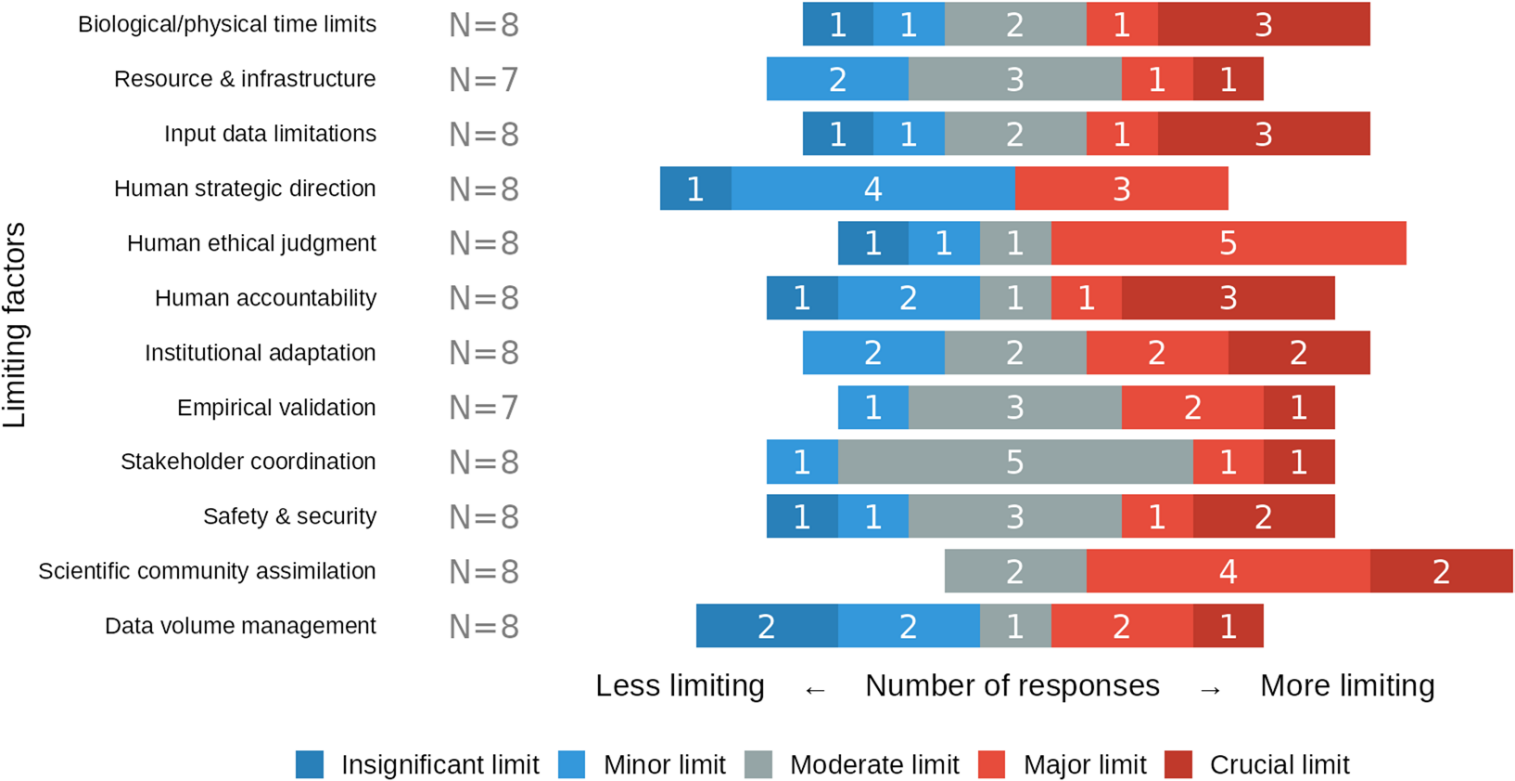
Perceived severity of factors that may limit GPAI-driven research acceleration. Colors encode response categories: insignificant limit (dark blue), minor limit (light blue), moderate limit (grey), major limit (light red), and crucial limit (dark red), with number of responses in white.

We asked experts to rate the **significance of various potential bottlenecks** (Fig. 6). While many factors showed a mixed response, scientific community assimilation was rated by all respondents as a moderate (2/8 responses), major (4/8) or crucial limit (2/8). In contrast, human strategic direction was seen as a lesser constraint, with a majority rating it as a minor (4/8) or insignificant limit. There was a marked consensus that stakeholder coordination is only a moderate limit (5/8).

In addition to the quantitative ratings, **experts provided general considerations** (Table S7), in which they highlighted irreducible biological and social constraints. One researcher noted that for their project, “the blood sampling of 200 individuals simply takes a definite time,” while another pointed to the “fundamental time-frame of the experiment (i.e. looking at 3 month effect after intervention).” The limitations of social processes were also stressed, “the speed of publication with peer review and also the response of the co-authors cannot be changed,” underscoring that institutional adaptation and human coordination remain important bottlenecks. Experts also emphasized practical challenges in system integration and the socio-economic barriers to adoption. One expert noted the difficulty of “interfacing of various output/input systems” and also pointed to the “Cost/Benefit ratio,” suggesting that the high upfront cost requires “phenomenal trust in results” (see Tables S4–S7 for full survey results and Information S8 for survey interface).

## Discussion

Our investigation, synthesizing findings from the scoping literature review, including concrete acceleration factors from 16 studies, and assessments from eight expert researchers, suggests that the availability and diffusion of highly capable GPAI systems might cause a fundamental transformation in how biomedical research will be conducted. The results of the exploratory expert elicitation support our research project life cycle’s time distribution between cognitive and physical tasks (73% versus 67% for cognitive tasks). While the experts expressed skepticism about extreme acceleration of experiment planning and execution, as well as hypothesis generation—suggesting that transformative research activities will continue to be constrained by human judgment and biological time constants—they considered high acceleration factors to be more plausible for structured processes such as manuscript preparation and the time-intensive process of publication. Most significant, however, is the unanimous concern about the scientific community’s ability to assimilate, suggesting that this could ultimately limit the pace of scientific progress.

### Limits to acceleration

While GPAI capabilities continue to advance rapidly, our analysis of the literature and expert feedback highlights several key factors that may limit the translation into overall research acceleration:

**Technical and infrastructural limitations**,** a recurrent theme in the reviewed literature**, encompass the technological prerequisites for GPAI-accelerated research and the shift from local to centralized cloud labs. These prerequisites include the availability, capacity and flexibility of cloud labs and automated devices, computing resources for training and operating GPAI systems, the capital expenditure and high fixed costs of self-driving laboratories, and the quality and accessibility of research data.

**Biological and physical limitations** impose fundamental boundaries on acceleration. Cell division rates, organism development cycles, or the duration of clinical trials follow natural timescales (as an expert noted the “fundamental time-frame of the experiment”, Table S7) that cannot be compressed beyond certain limits. Similarly, material handling and physical operations have speed limitations due to physical limits and safety considerations. However, we note that the growing effectiveness of in-silico simulation may affect the balance between fast computational experiments and slow wet-lab experiments.

**Institutional and regulatory factors** pose significant potential barriers to acceleration. As indicated by our expert panel, social processes, including ethical review, peer review, and publication, present particular challenges as they involve human judgment, predefined institutional procedures, and regulatory requirements that may not readily adapt to technological advances. The pace of GPAI adoption is significantly determined by the adaptability of institutions and workforces (as experts rated “scientific community assimilation” as the most significant limit, Fig. 6).

**Human oversight requirements** may remain indispensable for some aspects of research. Strategic direction continues to be determined primarily by humans, and ethical considerations require human review of certain decisions. Quality control often requires human expertise. The effectiveness of collaboration between humans and GPAI depends on the trust researchers have in GPAI outputs, and more broadly, the scientific community’s ability to assimilate and adapt to these new tools (which was identified by our experts as a crucial bottleneck) could ultimately limit the pace of scientific progress.

In light of these potential limiting factors, we argue that the full potential of GPAI-driven acceleration may only be realized when technological change is complemented by transformations of social processes, infrastructure, and governance frameworks.

### Transformation of research processes

**Review and publication processes**, like peer review and ethics approval, may face strong incentives to streamline and transform (experts rated structured administrative processes as most plausible for acceleration, Fig. 5). Our expert elicitation indicated that publication processes constitute a substantial fraction of the overall project duration (expert reported timelines, Fig. 4), and can be plausibly accelerated in a very substantive manner (Table 1). Building on this finding, we suggest that advanced GPAI systems could prove more effective than humans at aspects of reviewing, potentially checking protocol compliance and ethical considerations with greater reliability and comprehensiveness. This could shift review processes from sequential, time-intensive review cycles (whose duration stems from coordination challenges) towards more continuous and immediate monitoring. Consequently, human roles may shift from providing detailed reviews to general reflection, setting high-level standards, strategic oversight of GPAI, and handling complex cases, potentially requiring specialized skills for GPAI-human collaboration.

GPAI has various potential applications within the peer review process, from submission preparation and reviewer-paper matching to providing direct assistance with evaluation and formulating clear reviews^69^. GPAI feedback can supplement the scientific process, particularly during early manuscript development, though human review currently needs to remain the foundation of the review process^72^. Though GPAI can reduce reviewer burden in human-in-the-loop settings for many but not all cases, implementation risks like bias and potential misuse demand systematic study^73^.

We further propose that **metrics of scientific quality** may need revision, and the optimal balance between speed and thoroughness requires careful consideration. Existing incentives valuing perceived novelty and quantity over reproducibility could exacerbate the proliferation of unreliable findings, but GPAI might also enable the highly detailed and transparent documentation needed for reproducibility.

We anticipate that the **dynamics of goal-setting and exploration** in scientific inquiry may change fundamentally. Unlike human teams requiring lengthy onboarding, GPAI systems can be deployed immediately, reconfigured, or scaled to explore hypotheses without administrative delays, allowing a rapid switch of research directions. GPAI systems could bridge disciplinary boundaries by processing vast amounts of literature and facilitating novel connections between previously separate bodies of knowledge. In such a setting, steering research directions requires attention to potential biases in dominant GPAI models, which may influence which research areas receive attention. GPAI could lead to a homogenization of research approaches, which undermines the diversity of perspectives that has historically driven scientific innovation. Mechanisms to identify such biases and incentivize diverse approaches to problem-solving may be needed.

**Resource requirements** will become increasingly important. Infrastructure capacity, material and energy availability for continuous GPAI operations, and computing power and data access may emerge as critical limiting factors. Institutions unable to adapt to these new requirements may face significant competitive disadvantages.

### Policy implications

These projected shifts in the research landscape imply significant policy challenges, from resource allocation and workforce adaptation to safety and governance.

**Resource allocation.** Given the high infrastructural requirements identified, the extent to which different fields and institutions benefit from GPAI will depend on their access to capital, computational infrastructure, laboratory automation and frameworks for effective human-GPAI collaboration. GPAI-driven research acceleration risks increasing inequalities between institutions and political entities, which can lead to a concentration of progress and significant power imbalances. The resulting capture of intellectual property and market share could further entrench these inequalities, even if some institutions remain only slightly behind the cutting edge of GPAI-driven progress. To counteract these risks, we recommend that policymakers should proactively fund GPAI-driven research in two complementary areas: building self-driving laboratories that can flexibly execute diverse physical experimental tasks, and expanding access to frontier model capabilities and computational resources required for cognitive research tasks.

**Workforce adaptation.** GPAI may complement human researchers by shifting their focus to higher-level strategic direction, analysis and validation. Research organizations may therefore need to restructure to take full advantage of GPAI opportunities. This includes providing GPAI knowledge and skills to current and future scientists, including overseeing GPAI projects and critically interpreting their results, as well as updating incentive mechanisms to reward effective and transparent use of GPAI.

**Preventing misuse.** Care must be taken when applying GPAI to research on dangerous biological agents to avoid increasing dual-use concerns. Effective international coordination is essential, including common approaches to monitoring, harmonized standards, uniform transparency requirements and uniform ethical guidelines for both advanced GPAI and biological laboratory automation.

**Efficient governance.** Traditional governance structures struggle to keep pace with GPAI-accelerated science. This requires efficient frameworks that allow for continuous monitoring and rapid adjustments so that policies can be iteratively updated as new risks and opportunities emerge. Such frameworks may require updating research assessment methodologies and incentive structures to maintain scientific quality and to achieve best possible outcomes. Careful integration of AI tools into the governance processes themselves may help meet this challenge.

### Limitations of this study

Our study has several important limitations. The generalizability of our framework remains limited by significant variations in institutional structures and research practices across scientific fields and organizations. Biomedical subfields possess distinct characteristics that may lead to uneven acceleration potentials not fully captured in our estimates.

While our study focused on estimating the potential acceleration of established processes and research tasks, long-term transformation by GPAI would likely go beyond mere acceleration of existing processes and introduce new paradigms in research. In our worked example, once cognitive and physical tasks are maximally accelerated, the irreducible time constants inherent to lab experiments become the dominant bottleneck (3 out of 3.6 months). Paradigm-shifting strategies that we excluded—such as replacing in vivo/vitro experiments with in silico experiments or introducing AI-first prioritization to decrease the amount of required experiments—could reduce this remaining constraint and create a qualitatively new research dynamic. Assessing the feasibility, risks, and governance implications of such transformations is beyond the scope of our investigation. This boundary condition limits the gains we report but increases the robustness of our estimates by restricting them to accelerations that are evidence-based, plausible and relevant within the current research paradigm.

Our framework of major research tasks categorized into discrete cognitive and physical tasks, while providing analytical clarity, represents necessary simplifications that may obscure important research acceleration dynamics. Many biomedical research tasks are inherently hybrid or have intertwined components, with real research characterized by frequent iterative switching between processes rather than discrete, sequential phases. Additionally, while our sequential framework does not capture real-world task parallelization by multiple team members or project downtimes and delays, we believe that these opposing influences cancel each other out to a certain extent, so that the resulting inaccuracy remains low.

The projection of GPAI capabilities inherently involves substantial uncertainty. The interactions between accelerated research processes, institutional structures, and social systems introduce additional unpredictability. Our modeling approach necessarily simplifies these dynamics and does not account for feedback loops or emergent phenomena that could significantly influence real-world outcomes.

Current empirical evidence provides an incomplete foundation for robust predictions. While promising examples of GPAI-accelerated research are emerging, long-term and large-scale implementations are still scarce. Our literature review revealed considerable heterogeneity in reported acceleration factors and how they were determined. The adaptation rate of social and institutional processes to technological acceleration represents another significant unknown with limited historical precedent.

The expert elicitation introduces potential selection bias and the number of experts consulted was relatively low at eight individuals, which limits the robustness of the results. Nevertheless, this elicitation provides an initial assessment of the plausibility of our key assumptions and findings, and reveals the prevailing attitude of experts toward GPAI and its potential to accelerate research.

Despite these constraints, our multi-method approach establishes a foundation for elucidating the limits to biomedical research acceleration. While our current estimates contain high uncertainty, they do suggest that order-of-magnitude acceleration through GPAI implementation is plausible. By identifying key drivers and barriers to acceleration, this work offers valuable guidance for scenario planning and policy development in an increasingly GPAI-driven research landscape.

### Future work

Based on the limitations and opportunities identified in this study, we highlight several avenues for future research. Future research should prioritize the collection of empirical data from GPAI-driven laboratories to validate the theoretical acceleration estimates presented here, taking implementation costs and time for setup into account. Controlled experiments in augmenting scientific review processes with GPAI should be conducted. Given the significant constraints identified regarding scientific assimilation in our expert study, future work must focus on designing institutional frameworks and incentive structures that facilitate the effective and responsible integration of autonomous systems into the human scientific community, including scientific review. Finally, as biological time constants are the most difficult barrier to address in accelerated workflows, investigating the validity and scope of high-fidelity in silico simulations to replace specific in vivo experiments is another important field of research.

## Methods

To analyze the potential acceleration of biomedical research through GPAI, we employed a multi-faceted approach combining framework analysis, literature review, and expert feedback. This methodology enabled us to systematically evaluate both current capabilities and future potential while maintaining practical relevance.

**Framework development**: We began by analyzing existing conceptual frameworks related to GPAI capabilities and research automation. We identified and reviewed four relevant frameworks: DeepMind’s “Levels of AGI,” which differentiates between narrow and broad AI systems^35^; Society of Automotive Engineers’ “Driving automation systems,” which describes levels of interaction between humans and increasingly autonomous vehicles^36^; “AI agents for biomedical discovery,” which details increasing levels of AI agency in biomedical research^37^; and “Self-Driving Laboratories,” which maps the integration of software and hardware processes for increasing laboratory autonomy^38^. We synthesized concepts from these frameworks (i.e., general reasoning from AGI models and autonomy levels from engineering standards) to create a domain-specific framework for biomedical research that distinguishes between cognitive and physical capabilities (further described in the Results section). This integration was necessary because biomedical research uniquely requires high proficiency in both abstract reasoning and physical manipulation, which was not fully captured by any single prior framework.

**Research process mapping:** We systematically analyzed the biomedical research lifecycle to identify major research tasks and their subcomponents. We mapped the complete research process from initial knowledge synthesis to final publication, identifying nine major research tasks encompassing the full research cycle. We then decomposed each major task into constituent subtasks with distinct characteristics and classified tasks according to their primary capability dimension (cognitive vs. physical). This structured mapping provided a foundation for applying our capability framework and assessing acceleration potential across the research process.

**Scoping literature review**: We conducted a scoping review of literature on GPAI-driven research acceleration published within the last five years up to March 2025. Given the rapid pace of development in this field and the lack of standardized terminology, we employed an iterative search strategy rather than a fixed systematic review protocol. We searched major databases (PubMed, Google Scholar) and preprint servers (arXiv, bioRxiv) using combinations of keywords related to technology (e.g., “Large Language Model,” “Generative AI,” “AI Agent,” “Automated Lab”) and efficiency (e.g., “acceleration,” “speedup,” “throughput,” “efficiency,” “time reduction”). We supplemented this database search with forward and backward citation chains: the backward search involved reviewing the reference lists of important articles to identify foundational works, while the forward search used citation tracking tools to find more recent studies that cited seminal works in the field.

The primary inclusion criterion was the reporting of quantitative acceleration metrics (e.g., reduction in time or increase in throughput) for specific biomedical research tasks. Studies were excluded if they were purely theoretical, lacked concrete baseline comparisons, or focused solely on predictive accuracy without reporting efficiency gains. For each included study, we extracted the research tasks addressed, the GPAI technology or methodology employed, and quantitative acceleration metrics (e.g., time reduction, throughput increase). When studies reported acceleration factors as ranges, these were converted to point estimates using the arithmetic mean (e.g., a range of [150–300] becomes 225; see Table S3) to facilitate the quantitative analysis presented in Fig. 3. One paper reports 10x reductions for semiautomated methods and 1000x reductions in researcher time for manual methods. We report more conservative 10-100x reductions because we account for total experiment duration, not just researcher time, yielding approximately 100x overall acceleration (1000 experiments require 500 days manually versus 5.5 days autonomously—5 days experimental time + 0.5 days researcher time)^30^. We then mapped the findings to our framework, identifying task-specific acceleration potential, patterns across cognitive and physical dimensions, and current vs. theoretical acceleration limits. This approach allowed us to ground our acceleration estimates in empirical evidence while acknowledging uncertainty in projections of maximum capability levels, interpreting reported values as current upper limits while accounting for potential publication bias favoring optimistic estimates.

### Framework integration

We integrated findings from the literature review to develop our unified acceleration framework. We mapped acceleration metrics to specific research tasks and capability levels, complemented our findings using real-world research timeline examples, synthesized results into a concrete framework showing potential acceleration across different scenarios, and identified key bottlenecks and rate-limiting factors that might constrain overall research acceleration. This integrated approach ensured our framework was both theoretically sound and practically relevant, reflecting the current state of the literature while acknowledging the limitations of existing evidence.

### Expert elicitation

To complement our literature findings, we developed a structured questionnaire (see Information S8 for original survey interface) aimed at researchers with direct experience managing biomedical research projects. The elicitation process, deployed via the web-based platform Alchemer, was designed to capture real-world experience and professional judgment regarding potential research acceleration through advanced GPAI systems and their limitations. Given the novelty of the effects of GPAI, our goal was not to achieve statistical generalizability, but rather to capture the current sentiment and informed judgment of domain experts on the identified potential and limitations of GPAI in accelerating research.

#### Expert selection

We identified and contacted authors who published biomedical studies in high-impact journals (3x Immunity, 2x Nature, 1x Nature Genetics, 1x Allergy, 1x Cell Systems) between 2020 and 2025. Selection criteria ensured participants had led (as first authors) or coordinated (as last authors) the published research project. This approach targeted researchers with experience across the full research lifecycle from conception to publication.

#### Elicitation protocol and data analysis

To provide context and calibration, respondents were presented with a table summarizing acceleration levels reported in recent literature for each major research task, including relevant citations. This helped ensure estimates were grounded in current technological capabilities while allowing for informed projection of future potential. Building on this context, we structured our survey in four main sections:

1. **Project timeline estimation**: Respondents provided the total duration (in months) of a specific biomedical research project they had led or coordinated. They then estimated how time was allocated across the nine major research tasks we identified in our framework. This established a baseline of actual research timelines against which to assess potential acceleration. We calculated mean project durations and mean time allocation for each of the nine research tasks, then computed the proportion of time spent on cognitive versus physical tasks.
2. **Plausibility of acceleration estimation**: We presented a hypothetical future scenario defined by the availability and universal use of maximum-level GPAI - defined as future GPAI with autonomous decision-making, advanced multi-disciplinary reasoning, and deep integration with robotics. Based on our scoping review of recent literature, we provided respondents with estimated accelerations of ~ 100x for primarily cognitive tasks and ~ 25x for primarily physical tasks. For each of the defined nine major research tasks, respondents evaluated the plausibility of achieving these pre-defined acceleration factors in the context of their own project. Responses were collected on a five-point Likert scale ranging from “Significant Overestimate” to “Significant Underestimate”. Response frequencies were tabulated for each research task across the five-point scale, and we reported the number of respondents who rated each acceleration estimate as plausible, overestimated, or underestimated.
3. **Evaluation of limiting factors**: To identify key bottlenecks that might prevent achieving theoretical acceleration maximums, respondents were presented with a list of 12 literature-derived potential limiting factors. These spanned technical constraints (e.g., fundamental biological time limits), resource constraints (e.g., energy, infrastructure), and human/institutional factors (e.g., ethical oversight, regulatory adaptation). Respondents rated the significance of each factor in limiting practical acceleration on a five-point Likert scale from “Insignificant Limit” to “Crucial Limit”. We computed the distribution of ratings for each of the 12 limiting factors and identified factors with a unanimous or near-unanimous consensus among respondents.
4. **General considerations**: An open-ended section allowed respondents to share additional thoughts on research acceleration, potential risks, and ideas for mitigating bottlenecks. The responses were thematically analyzed to identify recurring themes related to implementation challenges, constraints, and opportunities for research acceleration.

## Supplementary Information

Below is the link to the electronic supplementary material.

Supplementary Material 1

## Data Availability

All anonymized elicitation data, analysis scripts and figure-generation code are available on GitHub: [https://github.com/OpenBioLink/ResearchAcceleration].
