# Supplementary material for: What are the limits to biomedical research acceleration through general-purpose AI?
Source: Sci Rep. 2026 Jan 12;16:1304. doi: 10.1038/s41598-025-32583-w (PMC12796276; doi:10.1038/s41598-025-32583-w)
Supplement: Supplementary file 1 — Supplementary Material 1 [file 41598_2025_32583_MOESM1_ESM.pdf]

Supplementary information

Supplementary Table S1. GPAI capabilities in research tasks

Major research task	Exemplary sub-tasks	Exemplary work
**Knowledge synthesis**	Finding & curating	(Skarlinski et al. 2024) Synthesizes scientific knowledge by retrieving relevant papers and summarizing their content in a cited, Wikipedia-style format. Outperforms human experts in precision and provides a more structured and accurate synthesis of scientific literature.<br>(Ghafarollahi and Buehler 2024) Knowledge graphs + agents can create novel hypotheses for science and rank them for novelty and feasibility.<br>(Z. Wang et al. 2022) Automated extraction of synthesis protocols from scientific literature to combat information overload through intelligent filtering and aggregation of research data.<br>(Luo et al. 2024) Automated literature search across multiple databases, standardization of research papers into experimental reports, and analysis of literature relevance and usability.<br>(Schmidgall et al. 2025) The PhD-agent retrieves relevant literature using the arXiv API. It uses iterative querying, evaluating abstracts, and full-text analysis to curate a set of high-quality research papers relevant to the research idea.
	Critical evaluation	(Elbadawi et al. 2024) Demonstrates critical thinking, predicting effects like laser scanning speed on printlet properties without prior precedent or templates in the literature.<br>(Swanson et al. 2025) Conducts critical evaluation through virtual team meetings involving multiple agents, including a dedicated Critic agent.<br>(Schmidgall et al. 2025) Assesses papers during literature reviews for relevance and importance, aligning findings with research goals and enabling automated summarization and cross-referencing for experimental planning.<br>(Ghafarollahi and Buehler 2024) Employs a Critic agent to review research proposals, highlighting strengths, weaknesses, and areas for improvement.
	Synthesize findings	(Swanson et al. 2025) Employs multiple AI agents collaboratively, led by a Principal Investigator agent overseeing project coordination.<br>(Schmidgall et al. 2025) Integrates curated literature into frameworks for experimental design and hypothesis development via human-agent collaboration or autonomous processing.<br>(Ghafarollahi and Buehler 2024) Expands hypotheses systematically, synthesizing findings into structured, comprehensive research outputs.
	Gap & contradiction identification	(Skarlinski et al. 2024) Detects contradictions in scientific literature by extracting claims, comparing them using a contradiction-detection prompt, scoring on a Likert scale, and validating with expert review.<br>(Ghafarollahi and Buehler 2024) Utilizes multi-agent systems and knowledge graphs to autonomously generate, critique, and refine hypotheses, identifying contradictions and gaps through structured data analysis and novelty assessment tools like the Semantic Scholar API.

**Idea & hypothesis generation**	Problem identification	(Ren et al. 2025) Generated hypotheses through text mining and multiple data sources, calculated success probabilities, identified TNIK as optimal target and progressed to Phase 2 trials.<br>(Schmidgall et al. 2025) Human researchers input a broad research problem, which the system refines into actionable goals. The PhD and Postdoc agents expand on this idea to create a structured research question, identifying specific opportunities for testing.<br>(Davies et al. 2021) Machine learning guides mathematical intuition and aids in faster discovery of new conjectures and theorems.<br>(H. Su et al. 2024) Multi-agent collaboration improves hypothesis generation quality, while reducing computing costs.<br>(Z. Liu et al. 2024) Specialized Agents produce testable hypotheses.<br>(C. Lu et al. 2024) The AI Scientist generates hypotheses, scores them for novelty and feasibility, and refines ideas using iterative chain-of-thought and self-reflection mechanisms.
	Hypothesis formulation	(Skarlinski et al. 2024) By flagging contradictions or evidence gaps these LLM-based literature agents can support the generation of new hypotheses.<br>(Ghafarollahi and Buehler 2024) AI uses graph reasoning to identify gaps, propose solutions, and ensure rigor and falsifiability through critique agents.<br>(Z. Liu et al. 2024) A 'falsification agent' verifies or refutes scientific claims by designing and executing automated ablation studies.<br>(C. Lu et al. 2024) Hypotheses are shaped into experimentally testable goals, ensuring alignment with research objectives.
	Theoretical framework development	(Ren et al. 2025) PandaOmics mapped pathways for lung fibrosis and cancer hallmarks, synthesizing literature into actionable solutions.<br>(Ghafarollahi and Buehler 2024) AI synthesizes knowledge graphs, links constructs, and provides mechanistic explanations, similar to PandaOmics.<br>(Z. Liu et al. 2024) Works within and refines theoretical frameworks through ablation testing and falsification to establish ground truths.<br>(Schmidgall et al. 2025) The agents build a framework around the research hypotheses, linking them to experimental design and objectives.<br>(Swanson et al. 2025) Inter-disciplinary agent meetings iteratively refine the project plan and tool chain.
	Feasibility assessment	(Ren et al. 2025) PandaOmics identified TNIK as the best target by analyzing pathways and calculating causal inferences.<br>(Ghafarollahi and Buehler 2024) AI evaluates hypotheses for novelty, practicality, and alignment with literature while proposing validation strategies.<br>(Swanson et al. 2025) Critique agents reviews practicality of code and methodological choices.<br>(Z. Liu et al. 2024) Experimental executability is ensured by translating proposals into structured, errorless instructions.<br>(Schmidgall et al. 2025) Automated troubleshooting and iterative refinement during experimentation ensure that research plans are executable.<br>(C. Lu et al. 2024): Assesses hypotheses for novelty and practicality, dynamically iterating on experiments to ensure executable research plans.

**Experiment design**	Method selection	(Ghafarollahi and Buehler 2024) AI agents systematically recommend tools and develop precise experimental and synthesis protocols.<br>(Swanson et al. 2025) Reasons across multiple non related disciplines (Biology, CS) to automatically design functional nanobodies using a computational pipeline (ESM, AlphaFold-Multimer, Rosetta).<br>(Schmidgall et al. 2025) The system formulates a detailed experimental plan, defining variables, objectives, methods and expected results. Plans are informed by literature review outputs and aligned with hypotheses to ensure reliable experiments.<br>(Rapp, Bremer, and Romero 2024) The AI autonomously designs experiments by predicting optimal protein sequences using Bayesian optimization, selecting candidates based on model predictions, and specifying experimental protocols tailored to test these hypotheses.<br>(Qu et al. 2024) CRISPR-GPT automates experiment setup by selecting CRISPR systems, designing guide RNAs, and tailoring delivery methods based on user objectives.<br>(Luo et al. 2024) The AI uses retrieval-augmented generation and hierarchical learning to analyze literature and datasets, identifying experimental frameworks and variables.<br>(C. Lu et al. 2024) The AI generates experiment designs based on initial templates, existing literature, and self-generated hypotheses. It plans experiments iteratively, incorporating feedback from results to improve designs.<br>(Zhou et al. 2024) AutoBA generates detailed, customized analysis plans by leveraging user-provided data paths, descriptions, and objectives.
	Protocol development	(Ghafarollahi and Buehler 2024) Proposes detailed experimental protocols in defined steps.<br>(Swanson et al. 2025) Develops protocols for automated ESM mutation analysis, AlphaFold structure prediction, and Rosetta calculations.<br>(Jiang et al. 2024) ProtoCode automates the curation and standardization of protocols from unstructured text.<br>(Schmidgall et al. 2025) AI designs protocols to run experiments autonomously, ensuring clarity and reproducibility.<br>(Qu et al. 2024) Generates detailed protocols, including gRNA design, delivery setups, and off-target prediction, while integrating external tools for resource optimization.<br>(Luo et al. 2024) The AI designs detailed protocols step-by-step, including headings, outlines, and experimental details based on analyzed data.
	Quality control	(Jiang et al. 2024) Standardizes protocols to ensure quality.<br>(Schmidgall et al. 2025) Automated error handling and iterative self-reflection ensure robust experimentation. Quality checks are integrated into every stage, including debugging and runtime error detection during data preparation.<br>(Ghafarollahi and Buehler 2024) Iterative feedback ensures protocols are robust and aligned with the hypotheses, including clear steps for modeling, synthesis, and testing.<br>(Qu et al. 2024) CRISPR-GPT provides validation workflows, such as sequencing, functional assays, and off-target analysis, to ensure experimental accuracy and compliance.<br>(Luo et al. 2024) The AI employs an LLM-based reviewer to ensure protocols meet quality metrics like completeness, correctness, and logical soundness.

**Ethics approval & permits**	Initial screening	(G. Singh et al. 2023) AI can enhance efficiency and standardization by rapidly analyzing documents and applying consistent criteria to identify potential ethical issues.<br>(Sridharan and Sivaramakrishnan 2025) LLMs can streamline ethics review processes by helping institutional review board members evaluate protocols more efficiently.<br>(Sridharan and Sivaramakrishnan 2024a) AI can speed up initial screening by quickly identifying good clinical practice violations and standard operating procedure deficiencies.<br>(Mann et al. 2025) An LLM can automatically review submissions for completion and ethics issues, suggest categories, and highlight where additional details are needed.
	Scientific review	(G. Singh et al. 2023) AI algorithms can provide a comprehensive perspective by exploring and cross-referencing databases of research studies and ethical guidelines.<br>(Sridharan and Sivaramakrishnan 2025) LLMs can systematically evaluate research proposals against guidelines and flag compliance issues, while complex ethical decisions require human expertise.<br>(Sridharan and Sivaramakrishnan 2024a) AI can enhance scientific rigor by assessing study design and eligibility for expedited review.<br>(Mann et al. 2025) LLMs can assist in scientific review by summarizing study aims, identifying key design elements, and highlighting ethical considerations.
	Ethics assessment	(Aydin et al. 2023) Physician-delivered informed consent was compared to an AI-based approach, which achieved better patient understanding while maintaining satisfaction levels.<br>(G. Singh et al. 2023) AI can assist in risk assessment and the review of the informed consent process, but requires human oversight to address complex issues.<br>(Sridharan and Sivaramakrishnan 2025) LLMs demonstrated ability to identify ethical issues in case studies but performed suboptimally in assessing placebo use, risk mitigation, and participant risks.<br>(Sridharan and Sivaramakrishnan 2024a) AI can provide a more consistent ethics review, but human oversight remains crucial.<br>(Mann et al. 2025) An LLM could provide a preliminary review, identifying ethical issues, precedents and guidelines, and a risk-benefit assessment.
	Regulatory compliance	(G. Singh et al. 2023) AI could facilitate transnational collaboration by supporting adherence to guidelines and regulations, using common tools and criteria across jurisdictions.<br>(Sridharan and Sivaramakrishnan 2025) AI can assist with adherence to guidelines by identifying missing elements and assessing fundamental ethical issues in research proposals.<br>(Sridharan and Sivaramakrishnan 2024a) AI can facilitate adherence to standards by drafting standard operating procedures, but human adaptation is needed.<br>(Mann et al. 2025) LLMs could support regulatory compliance by comparing protocols with applicable regulations, and institutional policies, also considering national or local context.
	Administrative processing and monitoring	(Aydin et al. 2023) AI streamlines the process of gathering and presenting information to patients, reducing clinical workload and costs.<br>(G. Singh et al. 2023) AI can automate administrative tasks, maintain documentation, and support continuous learning and adaptation to new findings.<br>(Sridharan and Sivaramakrishnan 2025) LLMs offer cost-effective solutions by generating initial drafts and training materials but still require human review and editing.<br>(Sridharan and Sivaramakrishnan 2024a) AI offers cost-effective solutions by streamlining standard operating procedure creation and assisting in the administrative tasks, requiring human validation.<br>(Sridharan and Sivaramakrishnan 2024b) LLMs show potential to automate patient instructions and materials, though successful implementation requires proper medical oversight.<br>(Mann et al. 2025) LLMs could perform consistency checks against past decisions, institutional policies, and also flag inconsistencies in documentation.

**Experiment execution**	Experiment preparation	(Szymanski et al. 2023) Robots automate the preparation of materials, precise measurement, and transfer to ensure experiments start with minimal human intervention.<br>(King et al. 2009) The system autonomously designs experiments by selecting yeast strains and preparing growth mediums based on hypotheses.<br>(Rapp, Bremer, and Romero 2024) The SAMPLE platform integrates automated workflows for assembling DNA, preparing reagents, and configuring robotic systems for protein engineering experiments.<br>(Bromig and Weuster-Botz 2023) AI handles the transfer of media and inoculum for serial passaging between bioreactors, ensuring precision and reducing manual effort.<br>(M Bran et al. 2024) Leverages LLMs to integrate chemistry tools for chemical discovery, synthesis planning, and reaction prediction.<br>(Huang et al. 2023) AI automates phenotypic data collection and colony isolation, reducing manual setup and standardizing processes.<br>(Y. Wang et al. 2018) Modular robotic systems integrate precise timing and automated instruments to streamline sample handling, ensure scalability, and deliver reproducible execution of protocols.
	Experiment execution	(Burger et al. 2020) Executes fully automated multi-step workflows, managing parallel setups of 16 samples with precision, achieving high acceleration in experimental processes.<br>(Jiang et al. 2024) Converts protocols into machine-readable formats for lab equipment.<br>(Szymanski et al. 2023) Robots in the A-Lab autonomously execute tasks such as synthesis, heating, cooling, and data collection, integrating physical actions with AI-driven decision-making to ensure precision, consistency, and minimal human intervention in experimental workflows.<br>(King et al. 2009) It uses laboratory automation to physically execute the experimental plan, including inoculating strains, managing growth conditions, and monitoring growth curves.<br>(Rapp, Bremer, and Romero 2024) The platform autonomously executes reproducible protocols like PCR amplification, thermostability assays, gene assembly, protein expression, and biochemical evaluations.<br>(Bromig and Weuster-Botz 2023) Real-time monitoring and automated consistent experiment execution accelerate processes, reducing time compared to manual methods.<br>(Huang et al. 2023) Robotic systems accelerate colony picking, imaging, and genotyping, improving experimental throughput.<br>(Y. Wang et al. 2018) The iQue PLUS Screener enables high-throughput data acquisition, reducing experiment runtime.
	Experiment documentation	(Luo et al. 2024) The AI retrieves, filters, and processes datasets and literature into structured reports that serve as input for protocol design.<br>(Burger et al. 2020) The system collects process data and can be monitored remotely, with data stored for analysis.<br>(Jiang et al. 2024) Standardizes how protocols are recorded.<br>(Szymanski et al. 2023) Implemented automated collection and storage of XRD patterns, synthesis conditions, and reaction outcomes through an integrated control system.
	Troubleshooting and optimization	(Burger et al. 2020) Incorporates 24/7 CCTV monitoring, remote error resolution, automatic alerts for stock levels and failures, and Bayesian optimization for outcome improvement.<br>(Szymanski et al. 2023) Created an active learning system that could optimize failed syntheses by suggesting improved reaction pathways, though still requiring human oversight for some failure modes.<br>(King et al. 2009) Adam's ability to cycle through hypothesis testing and refine its approach highlights its optimization capabilities.

		(Rapp, Bremer, and Romero 2024) The system iteratively refines its protein engineering process through Bayesian optimization, improving the experimental design based on feedback from previous results.<br>(Bromig and Weuster-Botz 2023) AI uses real-time data and soft sensors to optimize growth conditions and reduce lag phases.<br>(Huang et al. 2023) Morphological analysis ensures accurate colony identification, enhancing data quality and reproducibility.<br>(Y. Wang et al. 2018) Iterative system refinements and flexible automation configurations minimize cell loss and maximize workflow efficiency.
	Material management	(Burger et al. 2020) Manages the sample lifecycle, including preparation, analysis, and storage, using an organized rack system to maintain sample integrity.<br>(King et al. 2009) The system utilizes an automated freezer and manages sample storage as part of its workflow.<br>(Szymanski et al. 2023) Robotics enable secure, efficient sample handling and storage throughout the experimental workflow.
	Equipment maintenance	(Burger et al. 2020) Handles positioning calibration and battery management with automated systems.<br>(Huang et al. 2023) The system integrates biobanking with searchable databases, enabling efficient data storage and retrieval.<br>(Y. Wang et al. 2018) Automated systems use a modular robotic platform and integrated instruments to reduce manual intervention in sample preparation.
**Data analysis**	Data collection	(Schmidgall et al. 2025) Data preparation is automated using the ML Engineer agent, which writes and validates data processing scripts.<br>(Ghafarollahi and Buehler 2024) The AI system proposes detailed protocols for experiments and molecular simulations, including specifying the types of data to be collected, such as binding energies and self-assembly structures.<br>(Qu et al. 2024) The system designs validation protocols, including methods selection and the design of primers, to guide the collection of experimental outcomes.<br>(C. Lu et al. 2024) The AI autonomously gathers, processes, and organizes experimental results, generating visualizations and structured summaries for scientific use.
	Data cleaning and organisation	(Zhou et al. 2024) Automates preprocessing steps, including quality control, adapter trimming, and alignment.<br>(Z. Lu et al. 2024) DeepFlow performs an integrity check on input LMD files, automatically removes debris and doublets, and standardizes datasets for analysis, replacing manual preprocessing steps with automated workflows.<br>(Schmidgall et al. 2025) Data cleaning is automated during the preparation phase to ensure high-quality inputs for experiments. The system uses Python scripts to format and preprocess datasets.<br>(Qu et al. 2024) The system manages and validates user inputs, particularly when handling guide RNA designs and sequencing information.<br>(Swanson et al. 2025) The Virtual Lab AI agents creates an iterative loop of selection and improvement of most promising candidates.

	Statistical analysis and pattern identification	(Zhou et al. 2024) AutoBA eliminates traditional bioinformatics complexity by autonomously handling the entire analysis pipeline - from tool selection to code execution - requiring only the input data and desired analysis goal.<br>(Luo et al. 2024) Utilizes an LLM-based agent to design and execute dry lab experiments, automating bioinformatics analyses based on experimental protocols.<br>(Schmidgall et al. 2025) Employs an mle-solver to detect meaningful patterns and validate experimental results against clearly defined scientific objectives, ensuring robust outcomes.<br>(Z. Lu et al. 2024) Using clustering algorithms, the AI efficiently identifies and classifies normal and abnormal cell populations.
	Data visualization	(Z. Lu et al. 2024) The AI generates clear, multidimensional scatterplots for comprehensive and interpretable results.<br>(Swanson et al. 2025) AI agents used tools like AlphaFold-Multimer, Rosetta, and ESM that could later be used to produce high quality visualizations.<br>(C. Lu et al. 2024) The AI Scientist generates figures and plots as part of its experimental workflow using Python scripts.<br>(C. Singh et al., 2024) AI powers data analysis by transforming complex datasets into clear explanations and insightful visualizations, enabling reliable interpretation and extraction of key findings.<br>(Schmidgall et al. 2025) The AI models generated two figures within the set limit.
	Validation analysis	(Zhou et al. 2024) Ensures reliability of code execution and results through an automated error-checking mechanism (e.g., automatic code repair).<br>(Schmidgall et al. 2025) Outputs are validated by comparing experimental results to hypotheses and scoring their alignment. Scoring functions assess the quality and effectiveness of results.<br>(Swanson et al. 2025) The Virtual Lab AI agents employ bioinformatic software modules to score nanobody candidates.
**Results interpretation**	Results synthesis	(Ghafarollahi and Buehler 2024) The system employs multi-agent AI to critically analyze and interpret data from a knowledge graph, providing detailed outcomes and mechanisms for proposed hypotheses.
	Hypothesis evaluation	(Schmidgall et al. 2025) The agents discuss and evaluate whether the experimental results support or refute the hypotheses.<br>(Ghafarollahi and Buehler 2024) The system includes Critic agents that evaluate hypotheses against novelty and feasibility using tools like the Semantic Scholar API.
	Result contextualization	(Schmidgall et al. 2025) Agents outline the broader implications of the findings in the discussion section of the report, guiding future research directions.<br>(Ghafarollahi and Buehler 2024) The AI demonstrates contextual awareness and adaptability in hypothesis generation by dynamically integrating agent interactions, incorporating human feedback for refinement, and leveraging tools like the Semantic Scholar API to ensure novelty and relevance in scientific ideas.

**Manuscript preparation**	Methods documentation	(Elbadawi et al. 2024) The AI was able to write a full methodology section, with detailed experimental protocols.<br>(C. Lu et al. 2024) The AI Scientist documents the content of each plot, ensuring the saved figures and experimental notes contain all the necessary information for drafting the paper.<br>(Weng et al. 2024) AI generates logically coherent, domain-specific texts and simulated experimental protocols step-by-step.<br>(Zhou et al. 2024) AutoBA is a transparent and interpretable tool that allows bioinformaticians to easily document, modify, and customize its methods, streamlining the data analysis process.
	Results presentation	(Elbadawi et al. 2024) The AI effectively authored the results section, embedding scientific insights and methodologies, including experimental protocols.<br>(Schmidgall et al. 2025) The paper-solver generates an initial scaffold, dividing the manuscript into standard academic sections.<br>(C. Lu et al. 2024) The AI scientist motivates, explains, and summarizes results with complete visualizations.
	Reference management	(Skarlinski et al. 2024) The system enforces inline citations by requiring citation identifiers for each assertion and limiting citations to the provided context, thereby improving the completeness and accuracy of citations.<br>(C. Lu et al. 2024) The AI Scientist automatically searches for relevant papers using the Semantic Scholar API, integrates citations during the write-up, and generates a complete reference list.<br>(Weng et al. 2024) Outperforms the AI Scientist by citing more papers, enabling a deeper understanding of related work.<br>(Q. Wang et al. 2021) AutoCite demonstrates that reliable citation and context generation in academic papers is achievable by integrating semantic and structural insights.
	Figure and table preparation	(Elbadawi et al. 2024) The AI created believable and compelling analytical data, including plots and photo-realistic images of the subject matter.<br>(Schmidgall et al. 2025) The AI models successfully generated two figures, though their output was constrained to a maximum of two.<br>(C. Lu et al. 2024) The AI Scientist creates figures by leveraging automated tools such as Aider, an advanced LLM-based coding assistant, which edits plotting scripts and generates visualizations based on experimental results.
	Data sharing	(Schmidgall et al. 2025) Creates sharable code repository for reproducibility.<br>(C. Lu et al. 2024) Saves experiment results and logs in reproducible formats for collaboration and transparency.
	Writing and revision	(Skarlinski et al. 2024) Generates Wikipedia-style articles with cited summaries. While not explicitly described as manuscript preparation, this is closely related to creating structured, referenced scientific content.<br>(Elbadawi et al. 2024) Was able to write a full publication-ready manuscript on GPT-4 based on its own synthetically generated data, including tables and figures.<br>(Weng et al. 2024) CycleResearcher can generate fully structured research papers with clear methodological descriptions, deliver reviews with detailed feedback across multiple criteria, and consistently evaluate and refine work through iterative feedback.<br>(C. Lu et al. 2024) Refines manuscripts iteratively using self-assessment and simulated peer-review feedback.<br>(Schmidgall et al. 2025) The system produces complete, submission-ready academic reports adhering to NeurIPS formatting standards, refining manuscripts through iterative edits for clarity and coherence, with LaTeX compilation ensuring document integrity.

**Publication process**	Journal selection & submission	(Pividori and Greene 2024) AI-assisted writing tools can reduce the burden of formatting and stylistic requirements in scientific writing.<br>(Lin et al. 2023) AI could help with journal selection by automated scope-evaluation, potentially reducing desk rejections.<br>(Kuznetsov et al. 2024) AI could help generate publication metadata like keywords and track suggestions, and reformat submissions for different presentation styles.
	Screening & reviewer assignment	(Lin et al. 2023) AI tools can speed up manuscript screening by evaluating submissions for format, plagiarism, and article type.<br>(R. Liu and Shah 2023) LLMs like GPT-4 can be used to verify author-provided checklists with a demonstrated high accuracy covering topics such as theoretical results, experimental results, and code.<br>(Kuznetsov et al. 2024) Automated screening systems can evaluate manuscripts for formatting and policy compliance while optimizing reviewer assignments through improved keyword and content similarity matching.
	Peer review	(Lin et al. 2023) While AI-assisted systems will initially complement human reviewers, automated scholarly paper review could ultimately manage the entire evaluation process once challenges in data, parsing, interaction, and reasoning are resolved.<br>(R. Liu and Shah 2023) LLMs like GPT-4 have demonstrated error detection capabilities in scientific papers, identifying both mathematical and conceptual errors in test papers.<br>(Liang et al. 2024) AI reviewers provide paper-specific feedback, showing similar overlap with human reviews as found between human reviewers.<br>(Gao, Brantley, and Joachims 2024) LLM-generated reviews can be enhanced using aspect prompts to focus on specific parts of a paper, leading to detailed feedback that covers a range of opinions.<br>(Drori et al. 2024) Human judges rated LLM-generated reviews as comparable in quality to human reviews, though LLMs cannot yet handle all review cases independently.<br>(Weng et al. 2024) CycleReviewer demonstrates expert-level review capabilities, providing detailed, consistent feedback across multiple criteria and integrating iterative feedback throughout the research-review-revision cycle.<br>(C. Lu et al. 2024) The AI Scientist generates an automated review, according to current practice at standard machine learning conferences.
	Revision	(Pividori and Greene 2024) While the AI-based Manubot Editor successfully enhanced most paragraphs, some revisions introduced errors or omitted key information.<br>(Lin et al. 2023) Automated scholarly paper review can offer immediate feedback, enabling faster and more efficient revisions and improvements to manuscripts.<br>(Liang et al. 2024) LLMs can provide constructive feedback and suggestions for enhancing manuscripts, more than half of the users found the LLM generated feedback helpful.<br>(Drori et al. 2024) LLMs receive ratings equivalent to human reviewers in how helpful their reviews are at guiding authors toward paper improvements.

Supplementary Table S2. Research tasks vs. capability levels

Research task	No GPAI	Next-level GPAI	Maximum-level GPAI
**Knowledge synthesis**	Humans integrate all findings manually into cohesive frameworks.	GPAI merges intermediate findings, but humans lead the broader synthesis and narrative formation.	GPAI synthesizes multi-source data into frameworks, humans coordinate alignment to strategic goals or ethical standards.
**Idea & hypothesis generation**	All concepts come solely from human insight.	GPAI offers derivative ideas, suggestions or refinements; humans make the primary decisions.	GPAI proposes mostly novel, creative hypotheses with minimal human oversight; final acceptance may still require a human check.
**Experiment design**	All protocols are devised manually by humans.	GPAI suggests minor modifications to standard designs; major decisions require human approval.	GPAI autonomously creates optimized designs; humans primarily provide high-level guidance or ethical oversight.
**Ethics approval & permits**	Humans manually complete all documentation and navigate approval processes.	GPAI assists with form completion and provides guidance; humans manage key interactions and decisions.	GPAI autonomously prepares documentation and predicts approval requirements; humans verify and provide final sign-off.
**Experiment execution**	Humans conduct all procedures; no GPAI-driven instruments beyond basic machinery.	GPAI helps automate some repetitive tasks under human supervision; humans manage complex or critical steps.	GPAI-managed robotics carries out most tasks independently, with humans intervening only for specialized or ethical considerations.
**Data analysis**	Humans perform all data processing and analysis.	GPAI conducts intermediate-level analyses (e.g., trend identification); humans perform deeper contextual interpretation.	GPAI completes analyses, often revealing insights humans might overlook; final interpretation may require minimal human input.
**Results interpretation**	Humans derive and contextualize all conclusions.	GPAI highlights interesting patterns and generates preliminary conclusions; humans interpret the results.	GPAI formulates detailed interpretations with minimal human support; high-level human review remains a quality check.
**Manuscript preparation**	Humans write, organize, and edit manuscripts by hand.	GPAI helps draft manuscript sections (e.g., methodology) in close interaction with humans; GPAI refines language; humans structure the core narrative.	GPAI composes complete manuscripts, with humans offering only selective edits or high-level guidance regarding focus and style.
**Publication process**	Humans handle submissions, conduct peer review, and manage revisions entirely.	GPAI helps with formatting, requirement checks, and review response suggestions; humans conduct peer review and remain the main contact for publishers.	Separate GPAI systems autonomously manage the submission and review processes, handling all responses and revisions; humans review and give final approval.

Supplementary Table S3. Acceleration factors overview

Acceleration Range	Acceleration (Arithmetic Mean)	Task Type	Reference
1.3-2.0	1.65	Cognitive	BCG
2.0-4.0	3	Cognitive	Lu et al.
1.3	1.3	Cognitive	Dell'Acqua et al.
1.7	1.7	Cognitive	Noy & Zhang
2.2	2.2	Cognitive	GitHub Copilot
75-300	187.5	Cognitive	Skarlinski et al.
120-140	130	Cognitive	Zhou et al.
150-300	225	Cognitive	Luo et al.
1.7	1.7	Physical	Bao et al.
1-2	1.5	Physical	Rapp et al.
3-6	4.5	Physical	Rapp et al.
15-50	32.5	Physical	Rapp et al.
20	20	Physical	Huang et al.
12-36	24	Physical	Omidvar et al.
40	40	Physical	Burger et al.
10-100	55	Physical	Burger et al.
10-100	55	Physical	Delgado-Licona & Abolhasani
2	2	Physical	Emerald cloud lab
90	90	Physical	Emerald cloud lab
100	100	Physical	Arnold

Supplementary Table S4. Expert survey: Project timelines

Total project	Knowledge synthesis	Idea & hypothesis generation	Experiment design	Ethics approval & permits	Experiment execution	Data analysis	Results inter-pretation	Manuscript preparation	Publication process
87	5	5	5	0	30	19	5	12	6
72	2	2	2	2	24	8	8	6	18
80	2	3	5	3	19	21	2	5	20
80	3	8	6	6	18	9	6	6	18
66	2	2	12	0	18	6	6	8	12
118	6	6	4	4	36	14	14	6	28
40	1	1	1	2	8	12	5	4	6
36	2	3	2	1	6	10	1	10	1

Supplementary Table S5. Expert survey: Acceleration plausibility

Knowledge synthesis	Idea & hypothesis generation	Experiment design	Ethics approval & permits	Experiment execution	Data analysis	Results interpretation	Manuscript preparation	Publication process
Moderate Overestimate	Significant Overestimate	Moderate Overestimate	Moderate Overestimate	Moderate Overestimate	Moderate Overestimate	Plausible Estimate	Moderate Overestimate	Moderate Overestimate
Plausible Estimate	Moderate Overestimate	Significant Overestimate	Moderate Underestimate	Moderate Overestimate	Plausible Estimate	Moderate Overestimate	Plausible Estimate	Plausible Estimate
Moderate Overestimate	Moderate Overestimate	Moderate Overestimate	Plausible Estimate	Significant Overestimate	Plausible Estimate	Plausible Estimate	Plausible Estimate	Moderate Overestimate
Moderate Overestimate	Moderate Overestimate	Significant Overestimate	Plausible Estimate	Moderate Overestimate	Plausible Estimate	Moderate Overestimate	Plausible Estimate	Plausible Estimate
Plausible Estimate	Moderate Overestimate	Moderate Overestimate	Moderate Underestimate	Moderate Overestimate	Moderate Overestimate	Moderate Overestimate	Plausible Estimate	Plausible Estimate
Significant Overestimate	Plausible Estimate		Plausible Estimate	Moderate Overestimate	Plausible Estimate	Moderate Overestimate	Moderate Underestimate	Moderate Underestimate
Significant Overestimate	Moderate Overestimate	Moderate Overestimate	Plausible Estimate	Significant Overestimate	Moderate Overestimate	Moderate Overestimate	Moderate Overestimate	Moderate Overestimate
Plausible Estimate	Moderate Overestimate	Significant Overestimate		Plausible Estimate	Plausible Estimate	Moderate Underestimate	Significant Underestimate	Significant Underestimate

Supplementary Table S6. Expert survey: Limiting factors

Biological/ Physical time limits	Resource & infra-structure	Input data limitations	Human strategic direction	Human ethical judgment	Human account-ability	Institutional adaptation	Empirical validation	Stakeholder coordination	Safety & security	Scientific community assimilation	Data volume manage-ment
Moderate	Major	Crucial	Major	Major	Crucial	Crucial		Moderate	Crucial	Major	Crucial
Crucial	Moderate	Insignificant	Insignificant	Insignificant	Insignificant	Minor	Minor	Moderate	Insignificant	Major	Moderate
Crucial	Minor	Moderate	Major	Major	Crucial	Major	Moderate	Crucial	Minor	Crucial	Major
Insignificant	Crucial	Minor	Major	Major	Crucial	Minor	Moderate	Minor	Major	Major	Insignificant
Minor	Minor	Moderate	Minor	Major	Minor	Moderate	Crucial	Moderate	Moderate	Major	Insignificant
Crucial		Major	Minor	Minor	Moderate	Major	Moderate	Major	Moderate	Moderate	Minor
Major	Moderate	Crucial	Minor	Moderate	Minor	Moderate	Major	Moderate	Moderate	Moderate	Minor
Moderate	Moderate	Crucial	Minor	Major	Major	Crucial	Major	Moderate	Crucial	Crucial	Major

Supplementary Table S7. Expert survey: Open comments

Other limiting factors

Response 1: *Interfacing of various output/input systems - ideally no human in chain - likely requires a single system handling AI and robotics or very limited, highly standardised systems? Maybe I am thinking too non-AI like and a human-like, general intelligence operating available individual robot systems can immediately use a non-roboter lab better than a human. Cost/Benefit ratio - upfront cost to set up such a system requires phenomenal trust in results (i.e. monetization (or "academic currency")) or one ends up with a much weaker patchwork of 2nd choice systems that have downsides, don't interact etc with each other. Though similar to e.g. black powder's effect in warfare, I assume once it is established, it is pretty much needed to keep up/stay relevant. (Rated as: Moderate limit)*

\- - - - -

Response 2: *Fundamental Biological/Physical/Chemical Time & Measurement Limits: In my research, the blood sampling of 200 individuals simply takes a definite time, which can not be shortened due to ethical reasons and since blood drawing cannot be faster even with maximum AI. (Rated as: Crucial limit)*

\- - - - -

Response 3: *Fundamental time-frame of the experiment (i.e. looking at 3 month effect after intervention) and schedules of the research subjects. (Rated as: Crucial limit)*

General considerations

Response 1: *"revisions" was part of two categories Some of the categories have common parts (e.g. revisions could also be a part of "data analysis" and "results interpretation")*

\- - - - -

Response 2: *While AI might accelerate data analyses and presentation, the speed of publication with peer review and also the response of the co-authors cannot be changed....*

\- - - - -

Response 3: *The project I used as reference for making the time estimates was riddled with errors in project management, e.g. 12 months of experimental execution were spent on trying to optimize the wrong thing - similar to hitchhikers guide through the galaxy we might ask the wrong questions/invest in the wrong avenues. I am not sure if human-level AI (faster but same "intellect") would be able to help with that? Though if early adopters (of a robotics lab headed by AI) do this, the potential to "waste" money faster than a human lab (i.e. by running 24/7) is high. Human Accountability & Responsibility I currently answered as a crucial limit, but this is a societal decision, can we relent control? (Humans likely won't want to be responsible for black-box processes - i.e. be in a scapegoat position). I guess a significant benefit comes from running 24/7, being able to utilize logistics efficiently (machines constantly running, many projects in parallel). I.e. emerging efficiencies of "scale" by one/many general purpose AIs coordinating instead of humans. Regarding time savings, perhaps a usable estimator is the difference between an "old Mom-and-Pa bookshop" and Amazon? On the experimental side there is A LOT of logistical inefficiency in most labs. (I have not checked the provided references, I assume they consider these aspects.) Overall, I would not give a lot of weight to my predictions/estimations and expect to be wrong about most of them.*

Supplementary Information S8. Expert Survey: Interface (PDF Archive)

Expert Elicitation: Limits to accelerating research through future AI

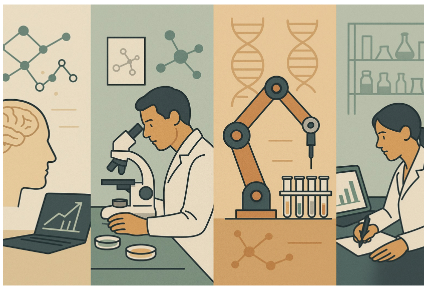

Introduction

"General-purpose AI" refers to AI systems that are capable of competently performing a wide range of distinct tasks. Rapid advances in such general-purpose AI, including powerful language models and sophisticated AI agents integrated with robotics and automated "cloud labs", are beginning to **reshape biomedical research**. Evidence suggests these technologies hold immense potential to accelerate scientific discovery.

General-purpose AI systems are demonstrating capabilities across the research lifecycle:

- **Cognitive tasks:** AI agents can rapidly synthesize vast amounts of literature, generate novel hypotheses, design complex experiments, analyze large datasets, and even draft manuscripts, sometimes achieving significant speedups (e.g., Skarlinski 2024a reported **100x** faster knowledge synthesis; Luo 2024a reported **150-300x** faster research cycles in specific contexts)**.**
- **Physical tasks:** Automated robotic systems and 'self-driving labs' enable high-throughput experimentation, parallel processing, and continuous operation, leading to substantial acceleration in areas like materials discovery and chemical synthesis (e.g., Burger 2020, Omidvar 2024 reported potential for 10-100x speedups; Arnold 2022 noted a 100x speedup in replicating a PhD project).

We analysed this emerging evidence and modeled potential acceleration under different AI capability levels. Based on documented achievements in specific, often optimized contexts, we derived plausible **upper-bound estimates for acceleration using future, highly advanced general-purpose AI systems ("Maximum-Level capabilities"):**

- **~100x acceleration** for primarily **Cognitive** research tasks.
- **~25x acceleration** for primarily **Physical** (experimental execution) tasks.

The hypothetical future scenario for this expert elicitation:

Please imagine the following — currently very futuristic — scenario:

1. **Maximum-level AI availability:** Very powerful general-purpose AI systems exist. They possess autonomous decision-making, advanced multi-disciplinary reasoning, and seamless integration with robotics. They can reliably plan, execute, analyze, interpret, and iterate on complex research cycles **with human-level capabilities and minimal human oversight, and at vastly increased speeds.**
2. **Universal access & use:** These powerful general-purpose AI systems are **accessible to everyone involved in the research process** – your group, collaborators, ethics committees, peer reviewers, journal editors, etc. They are striving towards delegating tasks where appropriate to achieve maximum effective acceleration while maintaining or improving quality.

Your input:

- Please consider the specific biomedical research project you have led.
- We will first ask about its actual timeline and task breakdown.
- Then, based on the hypothetical future scenario above, we want your expert judgment on the **realism** of achieving the **~100x (cognitive) / ~25x (physical)** maximum acceleration factors derived in our analysis, for each major research task within your project's context.
- We will also ask you to identify the key factors that you believe would limit acceleration, even with such powerful AI.

Your input will help better understand the true potential and practical boundaries of AI-driven research acceleration.
Thank you for your participation!

Section A: Your Past Research Project Timeline

Please think of **the specific biomedical research project** you substantially contributed to or led, from inception through to publication. Provide your best estimates for the questions below.

1. Estimated total project duration (in months): (From initial idea/synthesis to final publication)

[ ]

2. Estimated time allocation (in months) across major research tasks:
(Please ensure the months below add up to the total project duration entered above. These are estimates of primary focus time for your team) *

[ ] KNOWLEDGE SYNTHESIS (literature review, gap identification, etc)

[ ] IDEA & HYPOTHESIS GENERATION (problem formulation, feasibility, etc)

[ ] EXPERIMENT DESIGN (protocol development, methods selection, QC planning)

[ ] ETHICS APPROVAL & PERMITS (documentation, review process, compliance)

[ ] EXPERIMENT EXECUTION (lab work, sample prep, data acquisition, robotics)

[ ] DATA ANALYSIS (cleaning, statistics, visualization, bioinformatics)

[ ] RESULTS INTERPRETATION (synthesis of findings, hypothesis evaluation, context)

[ ] MANUSCRIPT PREPARATION (writing, figures, references, revisions)

[ ] PUBLICATION PROCESS (journal submission, peer review coordination, revisions)

Total : 0

Section B: Evaluating Maximum Potential Acceleration with Advanced AI

Now, consider your project again within the **hypothetical future scenario** described in the Introduction (maximum-level AI, universal access and use). For each task, evaluate how plausible our estimated maximum acceleration factor seems i**f your specific project** had been carried out in the hypothetical future scenario.

Task 1: Knowledge Synthesis

E.g., finding & curating information, critical evaluation, synthesizing findings, identifying gaps & contradictions

Our analysis suggests a potential maximum acceleration of **~100x** for this cognitive task with Maximum-Level AI.

How plausible is achieving this ~100x acceleration factor for this task in your project's context?

- ○ Significant Overestimate (Plausible acceleration likely much lower)
- ○ Moderate Overestimate (Plausible acceleration likely moderately lower)
- ○ Plausible Estimate (Stated acceleration seems plausible)
- ○ Moderate Underestimate (Plausible acceleration likely moderately higher)
- ○ Significant Underestimate (Plausible acceleration likely much higher)

Task 2: Idea & Hypothesis Generation

E.g., problem identification, hypothesis formulation, theoretical framework, feasibility assessment

Our analysis suggests a potential maximum acceleration of **~100x** for this cognitive task with Maximum-Level AI

How plausible is achieving this ~100x acceleration factor for this task in your project's context?

- ○ Significant Overestimate (Plausible acceleration likely much lower)
- ○ Moderate Overestimate (Plausible acceleration likely moderately lower)
- ○ Plausible Estimate (Stated acceleration seems plausible)
- ○ Moderate Underestimate (Plausible acceleration likely moderately higher)

- Significant Underestimate (Plausible acceleration likely much higher)

Task 3: Experiment Design

E.g., method selection, protocol development, quality control planning

Our analysis suggests a potential maximum acceleration of **~100x** for this cognitive task with Maximum-Level AI.

How plausible is achieving this ~100x acceleration factor for this task in your project's context?

- Significant Overestimate (Plausible acceleration likely much lower)
- Moderate Overestimate (Plausible acceleration likely moderately lower)
- Plausible Estimate (Stated acceleration seems plausible)
- Moderate Underestimate (Plausible acceleration likely moderately higher)
- Significant Underestimate (Plausible acceleration likely much higher)

Task 4: Ethics Approval & Permits

E.g., initial screening, scientific review, ethics assessment, regulatory compliance, administrative monitoring

Our analysis suggests a potential maximum acceleration of **~100x** for this cognitive task with Maximum-Level AI, assuming institutional processes also adapt.

How plausible is achieving this ~100x acceleration factor for this task in your project's context?

- Significant Overestimate (Plausible acceleration likely much lower)
- Moderate Overestimate (Plausible acceleration likely moderately lower)
- Plausible Estimate (Stated acceleration seems plausible)
- Moderate Underestimate (Plausible acceleration likely moderately higher)
- Significant Underestimate (Plausible acceleration likely much higher)

Task 5: Experiment Execution

E.g., preparation, execution, documentation, troubleshooting & optimization, material management, equipment maintenance

Our analysis suggests a potential maximum acceleration of **~25x** for this physical task with Maximum-Level AI and robotics/self-driving labs.

How plausible is achieving this ~25x acceleration factor for this task in your project's context?

- Significant Overestimate (Plausible acceleration likely much lower)
- Moderate Overestimate (Plausible acceleration likely moderately lower)
- Plausible Estimate (Stated acceleration seems plausible)
- Moderate Underestimate (Plausible acceleration likely moderately higher)
- Significant Underestimate (Plausible acceleration likely much higher)

Task 6: Data Analysis

E.g., data collection, cleaning & organization, statistical analysis, pattern identification, visualization, validation

Our analysis suggests a potential maximum acceleration of **~100x** for this cognitive task with Maximum-Level AI.

How plausible is achieving this ~100x acceleration factor for this task in your project's context?

- Significant Overestimate (Plausible acceleration likely much lower)
- Moderate Overestimate (Plausible acceleration likely moderately lower)
- Plausible Estimate (Stated acceleration seems plausible)
- Moderate Underestimate (Plausible acceleration likely moderately higher)
- Significant Underestimate (Plausible acceleration likely much higher)

Task 7: Results Interpretation

E.g., synthesis of findings, hypothesis evaluation, contextualization within existing knowledge

Our analysis suggests a potential maximum acceleration of **~100x** for this cognitive task with Maximum-Level AI.

How plausible is achieving this ~100x acceleration factor for this task in your project's context?

- ◯ Significant Overestimate (Plausible acceleration likely much lower)
- ◯ Moderate Overestimate (Plausible acceleration likely moderately lower)
- ◯ Plausible Estimate (Stated acceleration seems plausible)
- ◯ Moderate Underestimate (Plausible acceleration likely moderately higher)
- ◯ Significant Underestimate (Plausible acceleration likely much higher)

Task 8: Manuscript Preparation

E.g., methods documentation, results presentation, reference management, figure/table prep, data sharing, writing & revision

Our analysis suggests a potential maximum acceleration of **~100x** for this cognitive task with Maximum-Level AI.

How plausible is achieving this ~100x acceleration factor for this task in your project's context?

- ◯ Significant Overestimate (Plausible acceleration likely much lower)
- ◯ Moderate Overestimate (Plausible acceleration likely moderately lower)
- ◯ Plausible Estimate (Stated acceleration seems plausible)
- ◯ Moderate Underestimate (Plausible acceleration likely moderately higher)
- ◯ Significant Underestimate (Plausible acceleration likely much higher)

Task 9: Publication Process

E.g., journal selection & submission, reviewer assignment, peer review, revision, correspondence

Our analysis suggests a potential maximum acceleration of **~100x** for this (primarily) cognitive task with Maximum-Level AI, assuming institutional processes (journals, reviewers) also adapt.

How plausible is achieving this ~100x acceleration factor for this task in your project's context?

- ◯ Significant Overestimate (Plausible acceleration likely much lower)
- ◯ Moderate Overestimate (Plausible acceleration likely moderately lower)
- ◯ Plausible Estimate (Stated acceleration seems plausible)
- ◯ Moderate Underestimate (Plausible acceleration likely moderately higher)
- ◯ Significant Underestimate (Plausible acceleration likely much higher)

Section C: Evaluating Key Limiting Factors to Overall Research Acceleration

In Section B, we asked you to evaluate the plausibility of potential maximum acceleration factors (~100x for cognitive tasks, ~25x for physical tasks) derived from optimistic scenarios in the literature. Often, practical limitations prevent achieving such theoretical maximums.

This section explores potential reasons why the actual acceleration achieved, even with Maximum-Level AI, might fall short of those ~100x/~25x figures.

For each factor below, please rate how significantly it contributes to **limiting the practical acceleration** achievable for the relevant research tasks (cognitive or physical), potentially explaining why the maximum factors presented earlier might be difficult to reach or sustain in a real-world project context.

Use the following scale:

- **Insignificant Limiter:** Unlikely to prevent achieving the maximum stated acceleration.
- **Minor Limiter:** May slightly reduce the practically achievable acceleration below the maximum.
- **Moderate Limiter:** Likely causes a noticeable reduction from the maximum achievable acceleration.
- **Major Limiter:** Likely a significant reason why the maximum acceleration won't be achieved.
- **Crucial Limiter:** Likely a primary reason why the maximum acceleration is unrealistic in practice.

Please rate how significantly each factor limits the practical achievement of the maximum potential acceleration (~100x cognitive / ~25x physical) for the relevant tasks:

Fundamental Biological/Physical/Chemical Time & Measurement Limits

E.g., irreducible time for biological processes, chemical reactions, physical equilibration; fundamental limits on measurement speed/sensitivity

Insignificant Limit	Minor Limit	Moderate Limit	Major Limit	Crucial Limit
○	○	○	○	○

Resource, Energy & Infrastructure Constraints

E.g., availability, cost, supply chain, and total energy demands for materials, equipment, compute; time/cost for building/maintaining/upgrading labs/self-driving labs and compute infrastructure at scale

Insignificant Limit	Minor Limit	Moderate Limit	Major Limit	Crucial Limit
○	○	○	○	○

Input Data Limitations:

E.g., constraints from quality, quantity, accessibility, biases, or time needed to generate novel ground truth data for AI.

Insignificant Limit	Minor Limit	Moderate Limit	Major Limit	Crucial Limit
○	○	○	○	○

Human Strategic Direction & Goal Setting

E.g., need for human input to define research priorities, high-level goals, project scope, and criteria for "success"

Insignificant Limit	Minor Limit	Moderate Limit	Major Limit	Crucial Limit
○	○	○	○	○

Human Ethical Judgment & Value Alignment

E.g., necessity for human oversight for complex ethical decisions, societal value alignment, and assessment of broader impacts

Insignificant Limit	Minor Limit	Moderate Limit	Major Limit	Crucial Limit
○	○	○	○	○

Human Accountability & Responsibility

E.g., requirement for designated humans to hold ultimate responsibility for research conduct, outputs, and compliance

Insignificant Limit	Minor Limit	Moderate Limit	Major Limit	Crucial Limit
○	○	○	○	○

Institutional & Regulatory Adaptation:

E.g., delays caused by the time needed for institutions (universities, journals, regulators) to adapt processes, standards, and legal frameworks

Insignificant Limit	Minor Limit	Moderate Limit	Major Limit	Crucial Limit
○	○	○	○	○

Empirical Validation & Inherent System Unpredictability

E.g., time/resources for experimental testing of AI outputs; inherent unpredictability or complexity of the system under study necessitating iterative empirical cycles

Insignificant Limit	Minor Limit	Moderate Limit	Major Limit	Crucial Limit
○	○	○	○	○

Coordination & Consensus Among Human Stakeholders:

E.g., time delays from communication, deliberation, and agreement needed among essential human actors (collaborators, committees, editors, regulators)

Insignificant Limit	Minor Limit	Moderate Limit	Major Limit	Crucial Limit
○	○	○	○	○

Operational Safety, Security & Containment Assurance

E.g., time and procedures dedicated to ensuring the safe, secure, reliable, and contained operation of powerful autonomous research systems)

Insignificant Limit	Minor Limit	Moderate Limit	Major Limit	Crucial Limit
○	○	○	○	○

Scientific Community Assimilation & Conceptual Integration

E.g., limits on the rate the human scientific community can absorb, verify, conceptually integrate, and build upon a vastly increased volume of findings

Insignificant Limit	Minor Limit	Moderate Limit	Major Limit	Crucial Limit
○	○	○	○	○

Management of Extreme Data Volumes

E.g., potential bottlenecks in storage, transfer, curation, and accessibility arising from the sheer scale of data generated by hyper-accelerated research

Insignificant Limit	Minor Limit	Moderate Limit	Major Limit	Crucial Limit
○	○	○	○	○

Other Limiting Factors

If you believe other significant limiting factors are missing, please specify below

If you specified "Other" factors above, how significant a limit do you consider them, taken together?

(Skip if you did not specify "Other" factors)

Insignificant Limit	Minor Limit	Moderate Limit	Major Limit	Crucial Limit	N / A
○	○	○	○	○	○

Section D: General Considerations

Please use this space for any additional thoughts:

- Any uncertainties or confusion you had while answering.
- Broader perspectives on how AI might transform research (beyond just speed).
- Concerns or potential risks associated with highly accelerated, AI-driven research.
- Ideas for overcoming the bottlenecks you identified to facilitate responsible acceleration.

Section E: Contact Information

Name: *

Thank you for your time and valuable insights!
